# Genome-wide comparative methylation analysis reveals the fate of germ stem cells after surrogate production in teleost

**DOI:** 10.1186/s12915-024-01842-z

**Published:** 2024-02-16

**Authors:** Rigolin Nayak, Roman Franěk, Audrey Laurent, Martin Pšenička

**Affiliations:** 1 The University of South Bohemia in Ceske Budejovice, Faculty of Fisheries and Protection of Waters, South Bohemian Research Center of Aquaculture and Biodiversity of Hydrocenoses, Zatisi 728/II, 389 25 Vodnany, Czech Republic; 2 Department of Genetics, The Silberman Institute, The Hebrew University of Jerusalem, Jerusalem, Israel; 3 Fish Physiology and Genomics Laboratory, INRAE, Campus de Beaulieu, 35000 Rennes, France

**Keywords:** Surrogate production, Epigenetic remodelling, Germ stem cells, Transplantation, CpG methylation

## Abstract

**Background:**

Surrogate production by germline stem cell transplantation is a powerful method to produce donor-derived gametes via a host, a practice known as surrogacy. The gametes produced by surrogates are often analysed on the basis of their morphology and species-specific genotyping, which enables conclusion to be drawn about the donor’s characteristics. However, in-depth information, such as data on epigenetic changes, is rarely acquired. Germ cells develop in close contact with supporting somatic cells during gametogenesis in vertebrates, and we hypothesize that the recipient’s gonadal environment may cause epigenetic changes in produced gametes and progeny. Here, we extensively characterize the DNA methylome of donor-derived sperm and their intergenerational effects in both inter- and intraspecific surrogates.

**Results:**

We found more than 3000 differentially methylated regions in both the sperm and progeny derived from inter- and intraspecific surrogates. Hypermethylation in the promoter regions of the protocadherin gamma gene in the intraspecific surrogates was found to be associated with germline transmission. On the contrary, gene expression level and the embryonic development of the offspring remained unaffected. We also discovered MAPK/p53 pathway disruption in interspecific surrogates due to promoter hypermethylation and identified that the inefficient removal of meiotic-arrested endogenous germ cells in hybrid gonads led to the production of infertile spermatozoa.

**Conclusions:**

Donor-derived sperm and progeny from inter- and intraspecific surrogates were more globally hypermethylated than those of the donors. The observed changes in DNA methylation marks in the surrogates had no significant phenotypic effects in the offspring.

**Supplementary Information:**

The online version contains supplementary material available at 10.1186/s12915-024-01842-z.

## Background

Epigenetics plays a critical role in regulating the expression of genes during embryonic development and tissue differentiation in living organisms. The epigenome is defined as the structural adaptation of chromosomal regions [1], which maintains stable cellular processes in living individuals unless disrupted by external factors. The epigenetic modifications involve complex mechanisms governed by many structural and molecular regulators. The common mechanisms include (a) DNA methylation, (b) histone protein modification, and (c) non-coding RNAs [2]. Among all epigenetic marks, DNA methylation has been the most extensively studied [3]. The most common structural modification is the addition of a methyl group to the 5th carbon atom of a cytosine base, forming 5-methylcytosine (5-mC). The 5-mC modification in a DNA segment changes gene activity without changing the gene sequence [2, 4]. Hypermethylation in regulatory regions, such as promoters in the genome, is known to suppress transcription [5, 6]. In contrast, the loss of methylation marks, leading to hypomethylation, stimulates the expression of certain genes [7]. A methyl group-bound cytosine base is typically followed by guanine base in the sequence; this short segment is known as a CpG dinucleotide. In vertebrates, methylation most commonly occurs in the CpG context [8]. The percentage of methylated CpG sites varies by cell type [9] and individual organism. Any minor change to the epigenome can exert an impact on cellular function [10].

Surrogate production involves advanced reproductive biology-based biotechnology, which enables the production of donor-derived gametes via germline chimeras. Isolated germ stem cells (GSCs) from a donor species are implanted into the suitable sterile recipient (endogenous germ-cell-free individuals) to produce donor-derived gametes [11]. Surrogate production technologies have offered the possibility of preserving the germplasm of large-bodied commercially valuable species in smaller species with a shorter sexual maturation cycle [12, 13]. This technique provides a unique opportunity to cryopreserve maternal genetic information in fish, as neither fish eggs nor embryos can be cryopreserved. Therefore, there are efforts to develop techniques to conserve endangered species such as sturgeon [14–16]. Despite the recent advancements in this field, the practical application of this technology in aquaculture production is still questionable. Our understanding is limited to the morphological characteristics of the gametes and progeny produced via surrogacy. There are several questions pertaining to the reliability of this approach must be answered to maximize the possibility of applying surrogate production technologies in aquaculture. Epigenetic change is a major question associated with germ cell transplantation (GCT) that has never been reported. This mechanism is sensitive to internal and external environmental factors and can induce heritable phenotypical alterations [17]. Our knowledge of how and to what degree GCT can modify the epigenome (DNA methylation) of the donor germline is still scarce. Elucidating the epigenetic perturbations and their functional consequences will provide us insight into the reliability of the surrogate production approach.

Several factors may have a significant impact on the donor’s methylome during GCT. Donor GSC is subjected to systematic procedures and is exposed to a variety of chemical compounds during surrogate production. The complex gonadal tissue is then reduced to single cells and transplanted into recipients. Cell proliferation and differentiation of donor GSCs occur in the recipient’s gonad after transplantation, supported by the gonadal somatic cells of the recipient. Very few of the hundreds of transplanted cells survive to be incorporated into the recipient gonad. In a previous study, Saito et al. [18] reported the size difference of the larvae produced by surrogate in zebrafish. Franek et al. [13] observed different morphological characteristics in male and female donor-derived gametes. Therefore, we hypothesize that the methylome of donor GSCs may undergo alterations in the recipient gonad and may thus lose or gain some crucial methylation marks. Considering these reports, it is now crucial to think beyond the morphological characteristics of the gametes produced by the surrogate parent. The number of methylation studies on donor-derived gametes produced via GCT is considerably lower than any other epigenetic study. A study of spermatogonia transplantation in mice has been reported, and it described the methylation of donor-derived spermatozoa and their offspring, but the results were limited to only a few imprinting genes [19]. In vitro culturing and transplantation of foetal germ cells have been conducted in mice, and the derived offspring were reported to exhibit growth abnormalities due to differential methylation [20], whereas fresh testicular tissue grafting into the recipients led to no epigenetic abnormalities in the germ cells or the offspring [21]. However, no information on methylation alterations in donor-derived gametes in fish has been reported.

It was previously reported that in zebrafish (*Danio rerio*), DNA methylation pattern was preserved in the germline [22], and the paternal methylome was inherited by the embryos [23, 24], which is why it is crucial to decode the methylome of sperm produced by the surrogate. It has been shown that after endogenous germ cell depletion, all sterile zebrafish transdifferentiated into males [25, 26] and there was no female germline chimera after transplantation. Therefore, we used donor-derived sperm samples and progeny for this study. Sterilization to deplete the endogenous germ cells of the host species is a crucial step in surrogate production to prevent the production of recipient-derived gametes. Sterilization of a host can be achieved by chromosomal manipulation [27], gene knockdown, gene knockout [28], UV exposure [29], and chemical treatment [30]. In this study, we used vas::EGFP transgenic zebrafish as donors and two types of hosts: (1) dead end *(dnd)* gene knockdown (*dnd* gene is exclusively expressed in primordial germ cells (PGCs) and plays a crucial role in PGC migration to the gonadal ridge during embryonic development, *dnd*-morpholino (*dnd*-MO) prevents PGC migration by blocking the translation of dead end protein [31]; hence the recipient becomes sterile with no endogenous germ cells) and (2) zebrafish and pearl danio (*Danio albolineatus*) hybrids (cross-breeding between two closely related species interferes with homologous chromosomal pairing during meiosis or induces mitotic arrest [32]).

Zebrafish have been used in many studies to produce germline chimeras [18, 33, 34] and may possibly be used in the future to resurrect the germplasm of certain valuable strains because of their small size, ease of breeding, and short maturation period. Our aim in this study was to answer the following questions: (1) Do the donor-derived gametes from surrogates present a different methylation pattern than the donor? (2) What are the differences in the methylation pattern between two different surrogates transplanted with the same donor GSCs? (3) Do the progeny derived from surrogates inherit altered methylation marks? and (4) Is the surrogate production approach reliable considering the epigenetic changes? To gain a greater understanding, we generated 24 whole-genome methylome datasets of sperm and progeny from two surrogates and donors at single-base resolution using whole-genome bisulfite sequencing (WGBS). We thoroughly investigated the differentially methylated regions by comparing the same sample types to identify any alterations in all three groups. We explored promoter methylation and investigated the potential biological and molecular function disruptions in the germline that are inherited by progeny.

## Results

### Data from transplantation

**Fig. 1:**
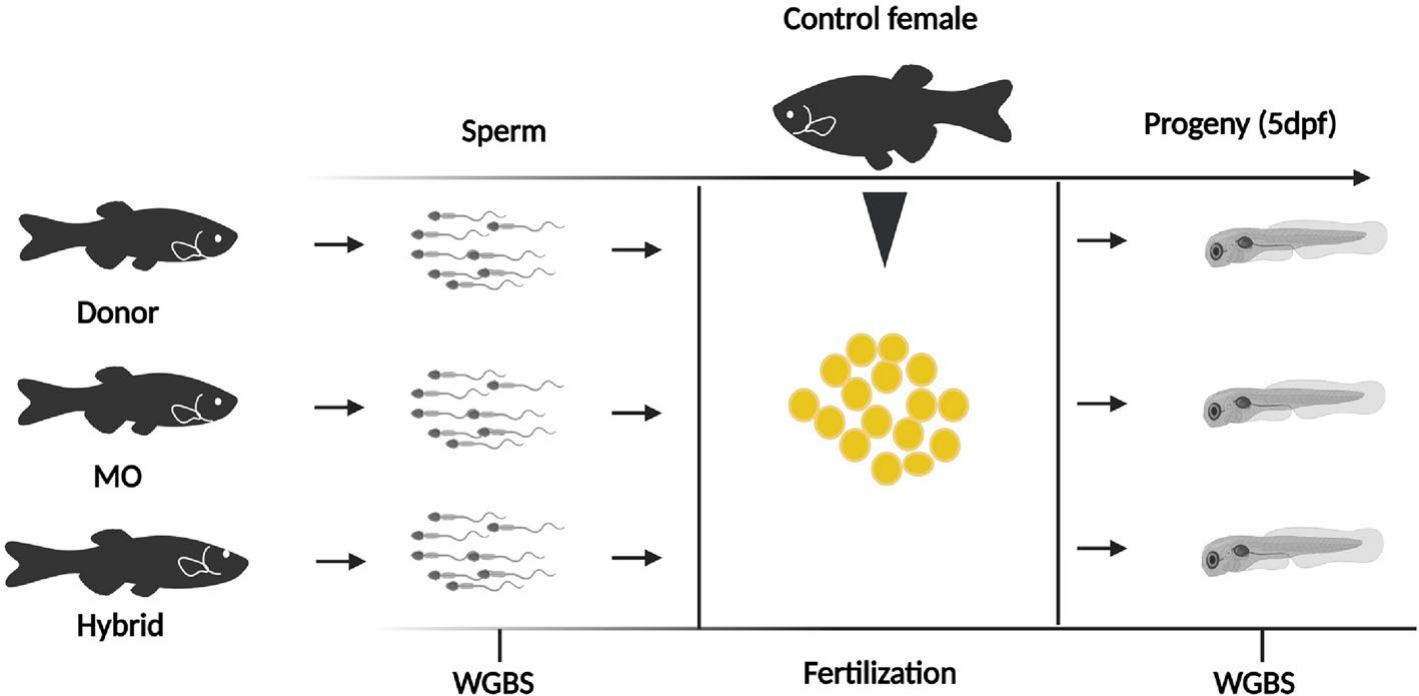
Schematic representation of the experimental designs. MO — morpholino-treated surrogate; dpf — day postfertilization. This figure was created with BioRender.com

Transplantation was performed in three replicates for MO and hybrid recipients at 5 dpf because hatchlings possess relatively immature immune system and transplanted spermatogonial cells are less likely to be immune rejected [35]. We screened the recipients at 14 dpt (day post-transplantation) to see the incorporation of donor spermatogonial cells and found an average of 45 and 35% of recipients were GFP-positive in MO and hybrid groups, respectively (Figure S1, Additional file 1). Upon maturation, an average of 40.4 and 21% of recipients from MO and hybrid groups produced donor-derived sperm (Table S1, Additional file 1), confirmed by GFP-specific PCR amplification, and no female germline chimera was observed in any group. Four adult germline chimera from each group were randomly selected for DNA methylation study (see Fig. 1 for the experimental design).

### Surrogate groups show a strong correlation with donors at the whole-genome methylation level

**Fig. 2:**
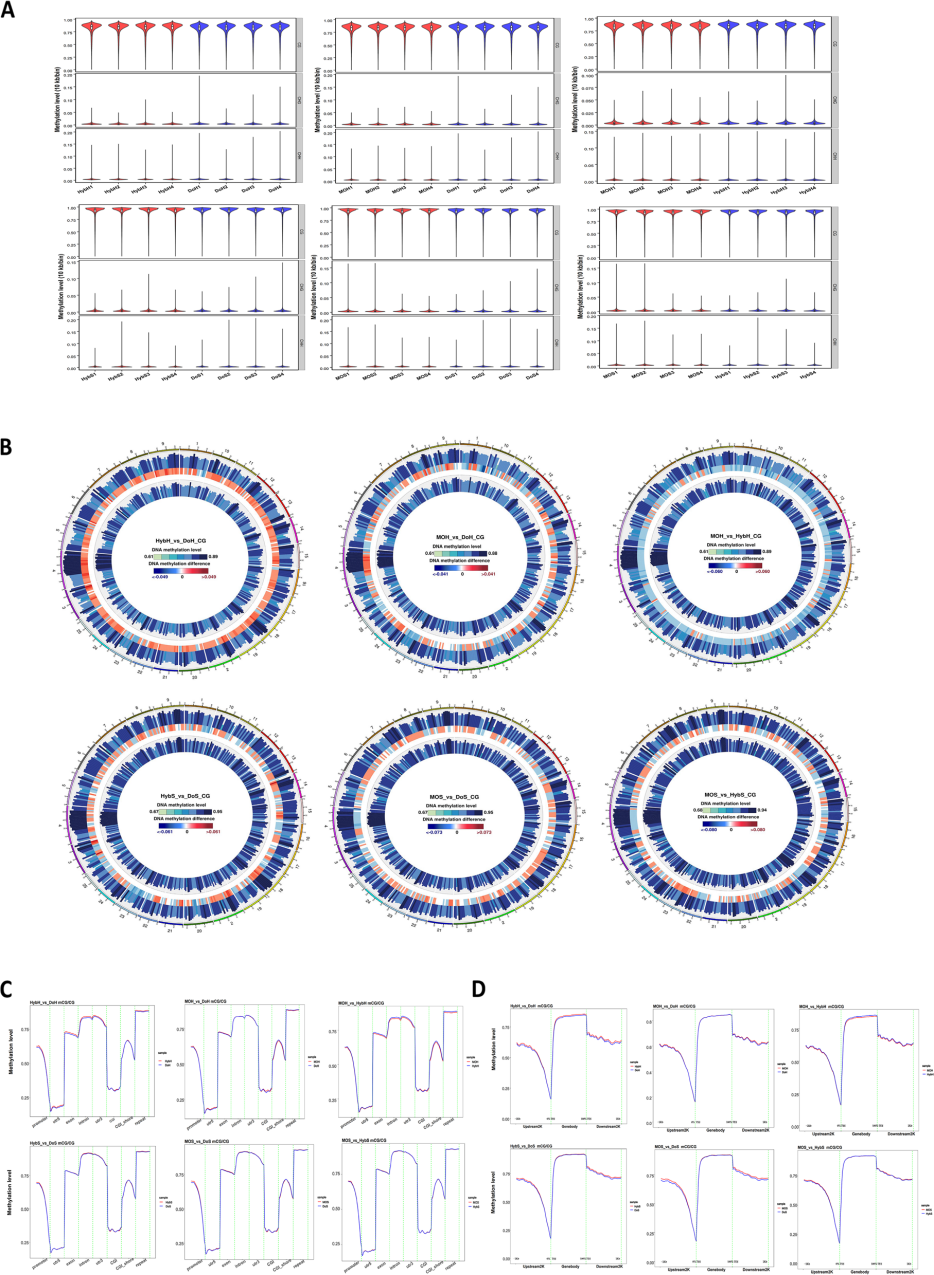
*Global methylation pattern in all three groups.* **A** Violin plots showing methylation levels in all three contexts. The whole genome was split into 10-kb sub-bins, and the methylation level was calculated for each context. The *x*-axis is labelled with the name of each sample, and the *y*-axis shows the methylation level. **B** Circos plots for methylation levels and their differences on each chromosome for the comparison groups. The chromosomes were divided into bins, and each bin’s methylation level was calculated as follows: number of reads with mCs/(number of reads with mCs + number of reads with non-mCs). Description of the circos plot from outside to inside: methylation level for Group 1, colour denotes the methylation level (%); methylation level difference between groups, and the heatmap shows the difference (%); methylation level for Group 2, colour denotes the methylation level (%) and graph legends. The upper panel is for the progeny samples, and the lower panel is for the sperm samples. **C** The methylation level in the functional regions (promoter, utr5, exon, intron, utr3, CGI, CGI-shore, and repeat elements). After combining the samples with biological replicates, each functional region was divided into 20-kb bins, and the methylation level of each bin was calculated. The upper panel is for the progeny samples, and the lower panel is for the sperm samples. Graph legends contain the compared group names. **D** Methylation level 2 kb upstream of the TSS, 2 kb downstream of the TES, and the gene body. The upper panel is for the progeny samples, and the lower panel is for the sperm samples. Abbreviations: MOS — sperm derived from the MO chimera; HybS — sperm derived from the hybrid chimera; DoS — sperm derived from the donor; MOH — progeny derived from the MO chimera; HybH — progeny derived from the hybrid chimera; and DoH — progeny derived from the donor, *n* = 4 biological replicates

We generated 24 whole-genome methylome datasets of ~ 30 Gb at single-base resolution for each sample (20 × depth for each), with 70% of bases being covered at least 5 times (5 ×) and 50% covered at least 10 × (genome coverage statistics is provided as Additional file 2). The bisulfite conversion rate was more than 99% for all the samples, with a mapping rate of approximately 60% of clean reads to the zebrafish reference genome GRCz11 (See Additional file 3 for mapping results). We calculated the average methylation level for all the genome cytosines in three different genomic contexts. We found that 87% of CpGs were methylated in the sperm samples for all three groups, and the average methylation level for the CHG and CHH contexts ranged between 0.35 and 0.4% (see Additional file 4, cytosine coverage in different contexts). In contrast, the progeny samples exhibited low CpG methylation levels, ranging between 79 and 81%, in which the CHH and CHG contexts accounted for 0.37 to 0.46%. When analysing the genome-wide methylation level, we did not observe a considerable difference between sperm and progeny samples in all three groups (Fig. 2A). Then, we examined methylation density at the whole-genome level. Methylation density showed high conservation among all groups and a trend similar to the methylation level, indicating a close relationship between methylation level and methylation density (Violin plots, Figure S2, Additional file 1). Since CHG and CHH methylation was negligible in all the samples, we focused only on CpG methylation in subsequent analyses.

Next, the correlation between replicates in each group was calculated using the Pearson correlation coefficient to determine closeness. The whole genome was divided into 2-kb bins, and the number of methylated cytosines (mCpG) was calculated for each bin. Replicates in each group showed a strong correlation, with *r*^2^ value > 0.92 (Figure S3, Additional file 1). Next, we checked the correlation between groups of similar sample types. We compared the sperm samples, which showed high homogeneity with *r*^2^ value > 0.91, and the result was similar to the progeny samples, in which *r*^2^ > 0.9.

### Differential methylation analysis suggests no massive epigenetic remodelling in the surrogates

Data from the replicates were combined for the differential methylation analysis between comparison groups at the whole-genome level (Fig. 2B, Circos plot). We observed that the hypermethylation rate in the HybH and MOH groups was slightly higher than that of the DOH group, and the difference in the methylation level was high among all three compared groups. Similarly, the sperm samples from all three groups showed higher differences with a different methylation pattern in all 25 chromosomes. Surprisingly, the landscape of CpG methylation in all genomic functional regions followed a very similar pattern with methylation depletion at the transcription start site (TSS) (Fig. 2C,D). Compared to those in other regions, introns and the repeat elements were hypermethylated and retained a similar shape in the plotted data for all the groups with no substantial difference. Compared to other genomic features, CpG islands (CGIs), which have been reported to predominate the promoter regions in vertebrates and to be involved in regulating gene expression [36, 37], were hypomethylated in all the samples and retained a similar methylation pattern in all studied groups. These results indicate that the sperm and progeny produced by the surrogates did not undergo a high degree of epigenetic remodelling.

### DMRs in the compared groups reveal hypermethylation in both surrogates

**Fig. 3:**
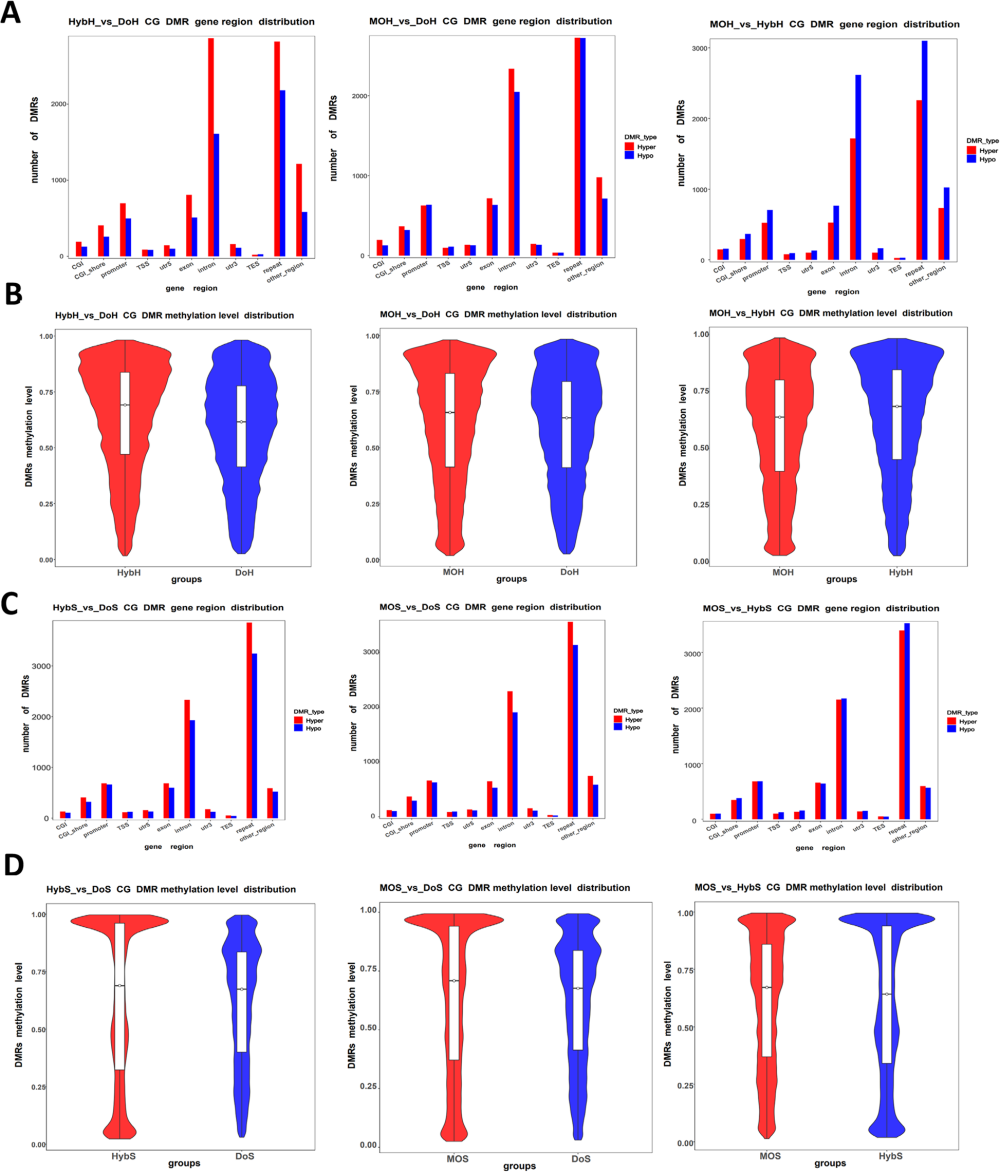
*DMR methylation level and their distribution in the functional regions*. **A** DMR distribution plot showing the different functional regions in the progeny sample comparison group. The *x*-axis shows the functional regions, and the *y*-axis shows the number of hyper-/hypoDMRs in each region. **B** Violin plot showing the DMR methylation level in the progeny samples in each comparison group. The *x*-axis shows the comparison group name, and the *y*-axis shows the methylation level. **C** DMR distribution in the sperm samples and **D** DMR methylation level of the sperm samples. The *x*-axis shows the comparison group name, and the *y*-axis shows the methylation level

Since we did not see any differences in the functional regions of the genome, we next investigated the differentially methylated regions (DMRs) in all groups. First, we calculated the number of DMRs in progeny samples across the genome. We found 3788 DMRs between the HybH and DOH, 3814 between the MOH and DOH, and 3766 between the MOH and HybH (Figure S4, Additional file 1). Then, we looked at hypomethylated (hypoDMR) and hypermethylated DMR (hyperDMR) in the compared groups. We annotated the DMRs according to their position in the genome (see Additional file 5 for the number of DMR distributions in all the genomic regions). More DMRs were found in introns and repeat elements than in other functional regions (Fig. 3A). When comparing HybH with DOH, we found more hyperDMRs in all the regions compared to the number of hypoDMRs. Then, we compared MOH and DOH and found almost the same number of both types of DMRs in all regions. However, the scenario was entirely different when we compared MOH with HybH, as we observed a higher number of hypoDMRs in all the regions, indicating higher methylation in HybH. Next, we calculated the methylation level of all the DMRs in the individual groups (Fig. 3B). The median value of the methylation level of the DMRs associated with the HybH was higher than that in the MOH and DOH. When we evaluated the DMR distribution with a violin plot, most DMRs were found to have accumulated with a higher methylation level in MOH and HybH.

Next, we asked whether the DMRs in sperm samples were similar to those in the progeny groups. To this end, we found 3639 DMRs between the HybS and DOS, 3527 between the MOS and DOS, and 3708 between the MOS and HybS. We annotated the DMRs, assessed their distribution (Fig. 3C), and calculated the methylation levels (Fig. 3D). The DMR distribution in all the sperm groups was similar to that in the progeny groups. The violin plots show that most of the DMRs in HybS and MOS groups exhibited higher methylation levels compared to those in the DOS group (also see the cluster heatmap of the DMRs in Figure S5, Additional file 1). The median value was higher, suggesting that sperm samples from both surrogates carried more hyperDMRs than the donors. These results indicate that both sperm and progeny derived from the surrogates were hypermethylated compared to the methylation level of the donors.

### Hypermethylation in promoters regions of protocadherin gene (*pcdh*) in MOH and HybS

**Fig. 4:**
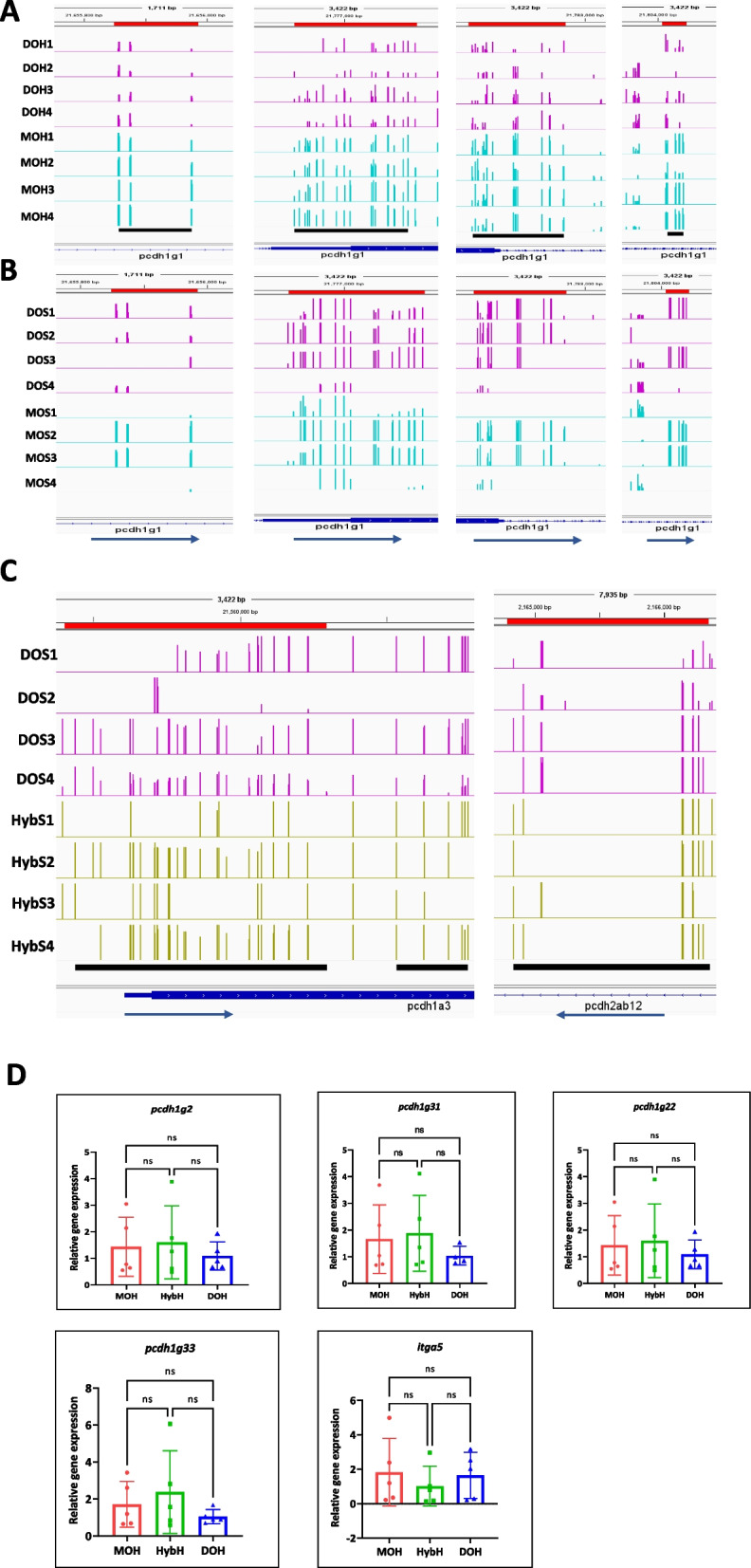
*Hypermethylation in the* *pcdh* *gene*. **A** IGV snapshots showing multiple representative hyperDMPs in *pcdh1g* gene, with increased methylation in MOH samples compared to DOH. **B** IGV view of the same promoter regions showing no hyperDMPs between the MOS and DOS groups. **C** Predictive hyperDMPs between the HybS and DOS groups in *pcdh1a* and *pcdh2a* genes. The black lines below the sample tracks represent predicted DMP regions. DMP regions are also highlighted by red lines above the sample tracks, and RefSeq gene annotations are shown below the tracks. **D** Relative gene expression levels of *pcdh1g2*, *pcdh1g31*, *pcdh1g22*, *pcdh1g33*, and *itga5* normalized to housekeeping gene *eef1a1l1* (Individual data values for qPCR are provided in Additional file 7). Data are shown as mean ± SD; One-way ANOVA followed by Tukey’s multiple comparisons for data with Gaussian distribution and Kruskal–Wallis test for data with non-Gaussian distribution (for *pcdh1g2* data), *p* < 0.05, *n* = 5 biological replicates, ns = not significant

Because most of the DMRs found were accumulated in intronic regions and repeat elements, we focused only on the promoter DMRs to see if any biological function was disrupted in the chimera groups (The list of significant DMPs between the compared group is provided as Additional file 6). Promoter regions are located within 1 kb from the TSS (− 1 to 0). Methylation in the immediate vicinity of a TSS hinders transcription initiation, whereas methylation in the gene body does not and may even stimulate transcription elongation [37]. Hence, we extracted all the differentially methylated promoters (DMPs) and analysed their GO terms; terms with *p*-value less than 0.05 were considered to be significantly enriched (Figure S6, Additional file 1). To our surprise, in the comparison groups for the progeny samples, more DMPs associated with biological processes were significantly enriched in the MOH compared to HybH and DOH. Biological functions such as homophilic cell adhesion, regulation of odontogenesis, and tooth mineralization were highly enriched in the MOH compared to HybH and DOH. We individually checked all the gene IDs for the promoters associated with this term in the Integrative Genome Viewer (IGV). The identified promoter regions encoded *pcdh1gc6*, *pcdh1g22, pcdh1g30, pcdh1g33*, *pcdh1g2,* and *pcdh1g31* (IGV Snapshots for *pcdh1g* DMPs, Fig. 4A). When visualizing individual replicates from each group of the progeny samples in IGV, the identified DMPs were found to be hypermethylated only in the MOH samples, and the pattern was consistent among the replicates. Next, we wondered whether these marks were present in the sperm samples. We compared the MOS and DOS groups and did not find significantly enriched DMPs related to these genes (Fig. 4B). When checking the methylation pattern in IGV for these DMPs, we see that HybS and DOS have similar methylation level, and the difference only arise in the progeny, where HybH and DOH show low methylation and MOH remains higher, suggesting that these loci in the HybH and DOH were demethylated while those in the MOH remained unchanged. These results indicated that hyperDMPs within the *pcdh* gene in MOH were transmitted through the germline.

We also found very few functions like cell–cell adhesion and homophilic cell adhesion enrichment for hyperDMPs in HybS, and the gene promoters targeted for these functions are *pcdh1g*, *pcdh2g*, and *pcdh1a* (Fig. 4C)*.* However, the hybrid progenies were unaffected by this hypermethylation since we did not see any function enrichment in the GO analysis. We measured the mRNA expression levels for selected genes, such as *pcdh1g2*, *pcdh1g31*, *pcdh1g22*, and *pcdh1g33* by RT‒qPCR (see Fig. 4D, individual data values for qPCR are provided in Additional file 7). However, We did not observe any significant difference in the gene expression level, which suggested that the hyperDMPs in *pcdh* did not affect the expression of these genes. Next, we analysed the hypoDMPs between MOH and DOH and found that functions such as regulation of angiogenesis, positive regulation of the developmental process, and positive regulation of vasculature development were significantly enriched. Angiogenesis is the process of blood and lymphatic vessel formation during embryonic development [38]. DMPs in genes *itga5* and *itga6l* were found to be hypomethylated. Integrin alpha 5 (*itga5*) is essential for heart development [39], but RT-qPCR showed no significant difference in the mRNA expression levels for *itga5*. Next, we examined the hypoDMPs in the comparison groups HybH and DOH for enriched GO terms. We found very few basic enriched molecular functions like nickel cation binding, adenylate kinase activity, nucleotide kinase activity, and phosphotransferase activity. The promoters associated with these terms in the progeny samples were predicted to have transcription factor activity. The genes are *si:dkey-14o6.4*, *si:dkey-8o9.5*, and *si:dkey-54j5.2* (complete list is provided as Additional file 8). These HypoDMPs were not significant in the sperm samples of any surrogate groups.

### Promoter hypermethylation in HybS reveals that MAPK/p53 pathway disruption leads to low-quality sperm production

Next, we investigated the KEGG (Kyoto Encyclopedia of Genes and Genomes) pathway enrichment for DMPs to determine whether any signalling pathway was disrupted. Pathways with a *p*-value lower than 0.05 in Fisher’s exact test were regarded as significant. When analysing the hypoDMPs between the MOH and DOH samples, we found that metabolic pathways, oxidative phosphorylation, and cardiac muscle contraction were highly enriched functional pathways (Figure S7, Additional file 1). The finding of cardiac muscle contraction enrichment was congruent with GO angiogenesis term enrichment in the MOH group. However, we did not find any significant pathway enrichment in the MOS group. Metabolic pathways are critical mechanisms, and oxidative phosphorylation is an essential function; enriched DMPs were found mainly in the mitochondrial genome (the complete list is provided as Additional file 9). Moreover, mitochondria are maternally inherited by progeny [40].

**Fig. 5:**
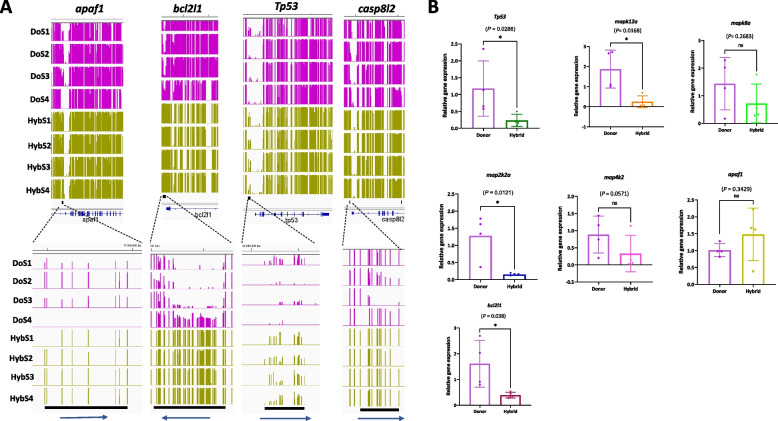
*Promoter hypermethylation in MAPK/p53 pathway genes*. **A** IGV snapshots of the representative hyperDMPs between HybS and DOS within the gene *casp*, *bcl2l1*, *Tp53*, and *apfa1*. Black rectangle and Refseq gene annotations are shown below the tracks. **B** Relative gene expression levels of *Tp53*, *mapk12a*, *mapk8a*, *map2k2a*, *map4k2*, *apaf1*, and *bcl2l1* normalized to housekeeping gene *eef1a1l1* (Individual data values for qPCR are provided in Additional file 7). Data are shown as mean ± SD; unpaired *t*-test for data with Gaussian distribution (*mapk12a*, *mapk8a*, *map2k2a*, and *bcl2l1* dataset) and the Mann–Whitney *U* test for data with non-Gaussian distribution (for *Tp53*, *apaf1*, and *map4k2* dataset) with adjusted *p* < 0.05, *n* = 4 biological replicates, asterisks represent significant difference between the groups, ns = not significant

Interestingly, we found that MAPK/p53 signalling pathway and apoptosis functions were significantly enriched in the HybS group, and these functional consequences were due to hyperDMPs in the HybS. We observed the promoter region of the *Tp53* gene in the HybS and found it to be hypermethylated compared to that in the MOS and DOS (see Fig. 5A). We found that the apoptosis pathway was also altered due to hyperDMPs in the genes caspase, apoptosis-related cysteine peptidase (*casp*), BCL2 like 1 (*bcl2l1*), and apoptotic peptidase activating factor 1 (*apaf1*). These genes are crucial for the activation of the apoptotic process. We then quantified the mRNA levels of selected MAPK/p53 pathway genes in the hybrid testes by RT-qPCR. A total of seven genes were quantified (*Tp53*, *mapk12a*, *mapk8a*, *map2k2a*, *map4k2*, *apaf1*, and *bcl2l1*), of which four genes *Tp53*, *mapk12a*, *map2k2a*, and *bcl2l1* showed a significantly lower level of gene expression compared to the donor (Fig. 5B).

In a previous comparison study by Franek et al. [41], donor-derived sperm from hybrid chimera showed low sperm velocity compared to that in the MO-treated chimeras, and the researchers speculated that the hybrids lacked a clearance mechanism. Nevertheless, the underlying molecular pathways were unknown. Our study revealed that promoter hypermethylation in hybrid potentially disrupted the function of MAPK kinases and the subsequent pathways, resulting in disturbed spermatogenesis. In our study, the in vitro fertilization rate of the donor-derived gametes from the hybrids with the control females was a deplorable success compared to MO and the donor males (Additional file 1, Table S2). Histological analysis of the untransplanted hybrid recipient gonad showed the presence of a few endogenous spermatozoa with developed testes (see Figure S8, Additional file 1). When analysing the sperm samples from the hybrid and MO chimera under the fluorescence microscope (Olympus fluorescence microscope), we observed the presence of spermatozoa without EGFP signal in the HybS. At the same time, the results from the MOS group were positive with EGFP expression in all the observed spermatozoa (See Figure S9, Additional file 1), which suggests that the semen samples of the HybS group are a mixture of both donor and recipient-derived sperm.

## Discussion

We thoroughly assessed the whole-genome methylome of donor-derived sperm from two types of surrogates transplanted with zebrafish spermatogonial cells to address potential DNA methylation remodelling caused by GCT at single-base resolution using WGBS and compared it with donor sperm. We also compared the progenies derived from the surrogate and donor and examined any potential biological and molecular function disruption by the gain or loss of methylome. The growing interest in surrogate production tools has been frustrated by a lack of knowledge on gamete features at the methylome level and the consequences of these features. Germline cells interact with gonadal somatic cells at every stage of development, which supports their growth, proliferation, and differentiation into functional gametes [42]. This interaction can be different for the foreign cells when introduced into another individual/species. Unlike genetic variation, epigenetic changes are more adaptable. When the environment changes, epigenetics changes more easily to adapt to the new environment. Some epigenetic changes are transient and can be reversed, while others are heritable and referred to as “ epigenetic memory” [43]. Surprisingly, our results showed high homogeneity among all groups of similar sample types at the whole-genome level and even in functional genomic regions. The lower percentage of methylated CpG in the progeny samples implies that the embryonic genome is activated after fertilization and is part of the essential developmental process [44]. Sperm methylome remains hypermethylated with no transcriptional activities due to its compact packaging and less accessibility to the transcriptional machinery. Global hypermethylation in both surrogate sperm and progeny is thought to be caused by the influence of the microenvironment, such as stress to donor testicular cells during isolation, and spermatogenesis in a recipient’s gonad may be factors leading to the genome-wide hypermethylation in the chimeras and inherited by their offspring.

Hypermethylation of *pcdh* promoters in the MOH was associated with germline transmission. Previous studies have reported that sperm methylation marks are preserved in the germline and are stably transmitted to the next generation [22]. The clustered *Pcdh* genes code for a group of homophilic cell adhesion proteins known as protocadherins, which are required for cell–cell adhesion during vertebrate embryogenesis. These proteins are also essential for neural development in zebrafish embryos [45–48]. However, after hatching, we did not observe any disturbed phenotype in the larvae (Figure S10, Additional file 1). *pcdh*- gamma hypermethylation has previously been reported in human brain tumour tissues to be involved in transcriptional repression [49]. DMRs in *pcdh* genes have been found in zebrafish after bisphenol A exposure [50]. However, there is no direct report on *pcdh* hypermethylation affecting any biological function in the teleost. One might argue if *pcdh* promoter methylations are from the oocyte origin. This question is answered with the DOH and HybH methylome produced using the same females. In zebrafish, promoter hypermethylation is not sure to be the repressive mark for gene expression. On the contrary, it induces expression for a few genes [44]. However, all the hyper- and hypoDMPs in the MOH group do not seem to affect the gene expression, as confirmed by quantitative PCR. These results imply that differential methylation does not always alter gene expression. The correlation between methylation and gene regulation varies among types of cells, genotypes, and under different environmental conditions [51, 52]. This phenomenon also opens the discussion to what degree of differential methylation drives the transcriptional changes and trait variability. Previous report suggests that methylation-dependent gene regulation activity is weak in human [53]. TALE-TET1 fusion proteins were used to induce target demethylation of CpG sites within the human *HBB* promoter, which revealed that demethylation of some CpG sites causally alters gene transcription. Demethylation did not affect *HBB* expression at other CpG sites in the same promoter [54], suggesting that CpG methylation-regulated gene expression is rather locus-specific for some genes to exert any expressional changes. Another study on zebrafish reported very little correlation between differential methylation and gene expression [55]. The relationship between differential methylation and gene expression is complex to be fully understood and requires extensive investigations.

Moreover, hybrid progenies were unharmed by the overall hypermethylation at the whole genome level because most DMRs were accumulated in the non-coding regions. Interestingly, our findings reveal the cause of low fertilization rate in the hybrid-chimera sperm due to discrepancies in the MAPK/p53 signal transduction pathway and apoptosis resistance in the recipient testes. Mitogen-activated protein kinases (MAPK) constitute the cascade of kinase proteins that control cell proliferation, differentiation, cell survival, and apoptosis through a signal transduction pathway in a coordinated manner [56]. Although we did not find altered gene expression levels in all the tested genes, we cannot rule out that *Tp53* transcript level was significantly lower together with other MAP kinases, *mapk12* and *map2k2a*, compared to the donor. p53 is a tumour suppressor protein believed to be activated after phosphorylation by MAP kinases. The activation of p53 further acts as a transcription factor for a group of genes that leads to the activation of the MAPK signal pathway [57]. Activation of p53 is also known to be involved in cell death and apoptosis. It is known that the MAPK pathway positively promotes the apoptotic pathway in the mice testis [58], which justifies the disruption of both pathways in HybS. In mammals, damaged germ cells are removed by apoptosis to prevent their differentiation into spermatozoa. Selective deletion of damaged germ cells is clearly an essential mechanism that protects a species genome [59]. A balance between the number of germ cells and Sertoli cells in the testis is also vital for the proper differentiation of germ cells and the production of fertile sperm. In the hybrid chimera, the damaged cells were the meiotic-arrested endogenous germ cells detected by the testis histology. The removal of damaged germ cells in the hybrid chimera was most likely suppressed due to apoptosis disruption caused by promoter hypermethylation of a group of genes, especially in the *Tp53* gene, resulting in an imbalance between the number of germ cells and Sertoli cells and the production of undernourished spermatozoa. The promoter region of the *Tp53* gene has been previously reported to be significantly hypermethylated in cervical cancer patients [60], oral squamous cell carcinoma [61], and the significance of apoptosis for the development of normal spermatogenesis is explained in mice [62, 63]. Deficiency in *Tp53* gene expression has also led to abnormal spermatozoa production and reduced fertility in p53 − / − mice [64]. These observed alterations in DNA methylation in the hybrids may have been caused by interspecific hybridization. Nevertheless, these functional disruptions were not transmitted to the progeny, possibly because the functional donor-derived sperm fertilized the oocytes. There is the possibility that the embryos fertilized with the epigenetically abnormal spermatozoa are not viable because they cannot undergo early embryogenesis [65].

As reported by Franek et al. [41], *dnd*-MO-treated recipients were completely devoid of endogenous germ cells upon maturation and carried only gonadal somatic cells. A complete germ cell-free gonad supports the incorporation and proliferation of transplanted exogenous GSCs without its competition with endogenous germ cells. In contrast, the hybrids were not entirely germ-cell-free, as apparent by the histology of the gonad. Low fertility in hybrid chimera is described in two scenarios: In one case, the inefficient removal of damaged endogenous germ cells is due to disruption in the apoptosis pathway, affecting the growth of the introduced GSCs and leading to the generation of low-quantity donor-derived spermatozoa. In the second case, the production of abnormal recipient-derived spermatozoa. Our findings infer that inter- and intraspecific spermatogonial transplantation in zebrafish surprisingly did not severely affect the molecular characteristics of the donor-derived gametes. However, the sterilization of the host plays a vital role in transplanted germ cell development in the recipient gonad. It is essential to ensure the complete depletion of the endogenous germ cell. Notably, isolated testicular cells maintained their methylome integrity after exposure to several chemical compounds and enzymes during cell dissociation. Previous studies have shown that spermatogonial stem cells are more resistant to epigenetic alterations and retain their stability after transplantation in mice [66]. The epigenetic integrity of in vitro cultured human spermatogonial stem cells has been reported [65], and that explains the germ stem cells are more protected against epigenetic alterations. We presume that there is a strong selection bias after their transplantation into the host, which explains the reason that only a few cells survive to colonize the host gonad. For interspecific transplantation, this scenario for methylome pattern may differ if the host is phylogenetically farther species, which needs to be investigated in the future. Our data cannot exclude the possibility that this scenario may vary from species to species and between different sterilization treatments. Zebrafish are highly polymorphic [67], and the methylation pattern can also differ between the individuals derived from the same parents. The advantage of using zebrafish as a model in this study is their lack of epigenetic reprogramming postfertilization. This makes them a suitable model to study epimutations caused by surrogate production and their intergenerational inheritance compared to other species like Japanese medaka, which undergo genome-wide reprogramming during embryonic development, and the majority of inherited marks are erased. However, whether the environmental stressor-induced DNA methylation marks escape the global epigenetic reprogramming in medaka is unclear. Our study is designed based on the most commonly used germ cell transplantation method. Gonial cell transplantation into larvae is easier than other methods, such as PGC transplantation. PGC isolation and transplantation are skill-sensitive and more complicated than gonial cell transplantation. Although PGC transplantation is well established in zebrafish, gonial cells are most commonly used for germ cell transplantation. There are several advantages of gonial cells over PGCs, including their abundance and transplantation into the hatchlings, which is considerably easier than PGC transplantation.

Moreover, we used freshly isolated testicular cells for transplantation in our study, and it will be interesting to understand the changes when transplanting the cryopreserved germ cells. Dimethyl sulfoxide (DMSO) is widely used as a cryoprotectant for the long-term storage of cell lines. In surrogate production techniques, cryopreserved germ stem cells are often used for transplantation. As per the previous study, DMSO significantly impacts genome-wide methylation in mouse embryonic cells and changes cell fate [68]. Future studies will explore the potential changes in the gamete produced by the transplantation of cryopreserved germ cells. The surrogate production technique is exploited to reduce the maturation period of some bigger species into fast-growing small fish [69, 70] and preserve the germplasm of endangered species [71].

## Conclusions

In summary, our study addresses the question of DNA methylation alteration in surrogate production and their persistence in the offspring by using teleost model species zebrafish. We report that (1) the genome of donor-derived sperm and progeny from both chimeras showed higher levels of methylation than the donor, and the observed hypermethylation in the surrogates did not cause any functional or phenotypical abnormalities in the produced offsprings, (2) protocadherin gamma was the most affected gene in the MO chimera by promoter hypermethylation and transmitted to the F1 progeny, (3) hypermethylated *pcdh*-gamma promoters did not affect gene expression or larval development in zebrafish and (4) low fertility in the interspecific hybrid was a result of apoptosis resistance in the meiotic-arrested germ cell. Our data sets the stage for further research on surrogates produced by evolutionary, more divergent species. Future studies will focus on the donor-derived gametes produced by the transplantation of cryopreserved germ cells.

## Methods

### Recipient production and transplantation

Adult AB line (wild type) zebrafish for recipient production were obtained from the European Zebrafish Resource Center (EZRC, Germany). Transgenic Tg(ddx4:egfp) (ZDB-TGCONSTRCT-210809–1, referred to as vas::EGFP) strain expressing enhanced green fluorescence protein in the germ cells driven by the promoter of the germ-cell-specific *vasa* gene [72], from the University of Liege, Belgium, and maintained in our facility for several generations at 28 ℃, 14/10-h light/dark photoperiod. All chemicals were procured from Sigma-Aldrich unless stated otherwise. The recipients were derived from the same parent (AB fish) and divided into two groups for *dnd*-MO injection (MO sequence 5′- GCTGGGCATCCATGTCTCCGACCAT-3′ [73], GeneTools, LLC) and hybrid production. *dnd*-MO (0.1 mM with 0.2 mM KCl) was injected into 1- to 2-cell-stage embryos. Hybrids were produced from AB females and pearl danio males as per the protocol described by Wong et al. [74]. Individual fish were first anaesthetized with 0.05% tricaine methanesulfonate (MS222). Then, the oocytes were collected from the ovulated AB females by applying gentle abdominal pressure, and milt was collected from the pearl danio (3-month-old) males; in vitro fertilization was then performed.

Testicular cell preparation from vas::EGFP donors and transplantation were performed according to the methods described by Franěk et al*.* [41] without any modification to the protocol. Three-month-old vas::EGFP males (*n* = 4) were anaesthetized with an overdose of MS222. Testes were removed and washed with phosphate-buffered saline (PBS) several times to remove the sperm and then fragmented into small pieces with a sterile scissor, followed by enzymatic digestion with 0.1% trypsin, 0.05% collagenase (Gibco) and 0.05% DNase in PBS for 60 min at room temperature (22–23 ℃). The digestion was then terminated by Leibovitz -15 (L-15) medium supplemented with 20% foetal bovine serum (FBS) at a 1:1 ratio. The cell suspension was filtered through a sterile 30-μm mesh filter (CellTrics) followed by centrifugation at 400 × *g* for 10 min. The cell pellet was then resuspended in L-15 medium containing 10% FBS.

Five-day-old recipients (from two groups) were anaesthetized with 0.05% MS222 and placed on a Petri dish coated with 1% agar. The spermatogonia cell suspension was loaded into a pulled-glass capillary connected to a micromanipulator (M-152, Narishige, Japan) and a FemtoJet 4 × microinjector (Eppendorf, Germany). The testicular cell suspension (500–600 cells, the number of sperm cells was not counted) was injected into the body cavity of each recipient. Fourteen days post-transplantation, recipients were evaluated under a fluorescence microscope (M205FA, Leica Biosystems) to detect the presence of GFP-positive cells. The individuals carrying GFP-positive cells were maintained at 28 ℃ in an incubator and fed with paramecium for 1 week and then with *Artemia nauplii* (Ocean Nutrition Europe). After 2 weeks in the incubator, they were transferred to a ZebTEC system (ZebTec Active Blue) and maintained until maturation with a dry diet (GEMMA Micro, Skretting) provided twice daily. Three months post-transplantation, milt samples from all the recipients were collected. Genomic DNA from the milt was extracted via the hotshot method [75] and validated with GFP-specific PCR primers (forward, 5′-ACGTAAACGGCCACAAGTTC-3′; reverse 5′-AAGTCGTGCTGCTTCATGT-3′) [76] using PPP master mix (TopBio). The amplification conditions were 94 °C for 5 min and 35 cycles of 94 °C for 30 s, 58 °C for 30 s, and 72 °C for 45 s, with a final extension of 72 °C for 5 min. The individuals showing positive amplification for GFP were considered to be germline chimeras.

### DNA extraction and WGBS library preparation

Sperm from the vas::EGFP donor males and surrogate males (*n* = 4, collected separately, three months old) were collected by stripping for each replicate individually according to the experimental design. Eggs from ovulated vas::EGFP females (3-month-old) were collected (*n* = 4, pooled) and divided according to the number of replicates in each group. Progeny was generated for each group by fertilizing the eggs separately for each replicate. For the donor group (control), sperm samples were collected from males (DoS1, DoS2, DoS3, and DoS4) and their progeny, which were the newly hatched (5 dpf) larvae (DoH1, DoH2, DoH3, and DoH4). Similarly, for the MO-treated surrogates, sperm samples were collected to establish four replicates (MOS1, MOS2, MOS3, and MOS4), and the progenies were established groups named in series from MOH1 to MOH4. For hybrid chimeras, the sperm samples were established for groups named in series from HybS1 to HybS4, and the progenies were in groups named in series from HybH1 to HybH4. Each male in all three groups was considered to be one biological replicate. Hatched (5 days old) larvae and sperm samples were processed for genomic DNA extraction using PureLink™ Genomic DNA Kit (Thermo Fisher Scientific). The concentration of the extracted DNA was measured using a Qubit fluorometer (Qubit 4 Fluorometer, Invitrogen), and the quality to know the integrity of the DNA was checked using 1% agarose gel. Accel-NGS Methyl-Seq DNA Library Kit (Swift Biosciences) was used for WGBS library preparation. Firstly, the genomic DNA spiked with unmethylated lambda DNA (for calculating the bisulfite nonconversion rate) was fragmented into 200–400 bp. During the bisulfite conversion, unmethylated cytosine was converted into uracil, while methylated cytosine remained unchanged. Methylation sequencing adapters were ligated, followed by double-strand DNA synthesis. The library was subjected to size selection (library size of 300 bp) followed by PCR amplification and final purification. The purified libraries were sequenced on the Illumina Novaseq platform with PE150 sequencing strategy (Novogene, Beijing, China).

### Histology of gonads

Histology was performed to evaluate the sterility of non-transplanted recipients. Three-month-old sterile recipients and intact controls (*n* = 3 for each group) were anaesthetized with an overdose of MS222 and carefully degutted. The whole torso was fixed with Bouin fixative for 24 h, followed by dehydration with a series of ethanol dilutions, embedded in JB-4 resin (JB4 embedding kit), using a plastic mold, and cut into 5-µm sections with a rotary microtome (Leica Biosystems). The sections were stained with hematoxylin and eosin according to the method described by Sullivan-Brown et al. [77]. Stained sections were then imaged under the Olympus microscope (Olympus BX51) and analysed to identify germ cells.

### RT‒qPCR for hyper- and hypoDMP genes

Total RNA was extracted from the larvae (5 dpf, *n* = 5 biological replicates) and the testis (*n* = 4 biological replicates) using a PureLink RNA Mini Kit (Thermo Fisher Scientific), followed by cDNA synthesis using WizardScript™ RT FDmix (Wizbiosolutions). Synthesized cDNA samples were used as templates for RT-qPCR. PCR was performed with PowerTrack SYBR Green Master Mix on a QuantStudio™ 5 System (Applied Biosystems), and the PCR cycling conditions were as follows: 95 ℃ for 2 min, 40 cycles (95 ℃ for 15 s, and 60 ℃ for 60 s) followed by a dissociation curve. The housekeeping gene eukaryotic translation elongation factor 1 alpha 1 (*eef1a1l1*) was used as the reference gene to normalize the expression of target genes. The primer sequences used for RT-qPCR are presented in Additional file 10. The level of gene expression was calculated using the 2^−ΔΔCT^ method. Four to five biological replicates were established for each group, and two technical replicates for each sample were established for RT-qPCR.

### Raw data processing and mapping to the reference genome

Before analysis, the reference data for zebrafish, which included the reference sequence fasta file, the annotation file in gtf format, the GO annotation file, the description file, and the gene region file in bed format, were prepared. As for the bed files, we predict repeats through RepeatMasker (version 4.1.2), followed by getting CGI track from the genome using cpgIslandExt. Bismark software (version 0.16.3, [78]) was used to perform alignments of bisulfite-treated reads to the zebrafish reference genome GRCz11. The reference genome was first transformed into a bisulfite-converted version (C-to-T and G-to-A) and then indexed using bowtie2 [79]. Sequence reads were also transformed into fully bisulfite-converted versions (C-to-T and G-to-A conversions) before they were aligned to similarly converted versions of the genome in a directional manner. Sequence reads that produced the best unique alignment from two alignment processes (the original top and bottom strand) were then compared to those from the normal genomic sequence, and the methylation state of all cytosine positions in each read was inferred. The reads that aligned to the same regions of the genome were regarded as duplicated reads. The sequencing depth and coverage were summarized using deduplicate reads. The results of methylation extraction (bismark_methylation_extractor) were transformed into bigWig format for visualization using the IGV browser. The sodium bisulfite nonconversion rate was calculated as the percentage of cytosine residues sequenced at cytosine reference positions in the lambda genome.

### Methylation level estimation

The sequence was divided into multiple bins of size 10 kb for the methylation level calculation. The sum of the methylated and unmethylated read counts in each window was calculated. The methylation level (ML) for each window or C (cytosine) site shows the fraction of methylated cytosines (mC) and is defined as Eq. 1. The calculated ML was further corrected on the basis of the bisulfite nonconversion rate as described in a previous study [80]. Given the bisulfite nonconversion rate “r”, the corrected ML was estimated as Eq. 2.

1 $$ML\left(C\right)=reads \left(mC\right)\div reads \left(mC\right)+reads (C)$$

2 $$ML\left(corrected\right) =ML-r\div 1 -r$$

### DMR analysis

Differentially methylated regions (DMRs) were identified using DSS software [81–83]. The core of DSS is a new dispersion shrinkage method for estimating the dispersion parameter from Gamma-Poisson or Beta-Binomial distributions. According to the distribution of the DMRs throughout the genome, the genes related to DMRs were identified as genes with a gene body region (from TSS to TES) or promoter region (upstream 2 kb from the TSS) that overlaps with a DMR. The DMR filtering command and parameters are as follows: smoothing = TRUE, smoothing.span = 200, delta = 0, p.threshold = 1e-05, minlen = 50, minCG = 3, dis.merge = 100, pct.sig = 0.5. Gene Ontology (GO) enrichment analysis of genes related to DMRs was implemented with the GOseq R package [39], in which gene length bias was corrected. GO terms with corrected *P* values less than 0.05 were considered to be significantly enriched. Kyoto Encyclopedia of Genes and Genomes (KEGG, http://www.genome.jp/kegg) is a database resource for understanding high-level functions and utilities of the biological system [84]. We used KOBAS software [39] to test the statistical enrichment (*P* < 0.05) of DMR-related genes in KEGG pathway**s**.

### Statistical analysis

Data for relative gene expression was first checked for normal (Gaussian) distribution with the Shapiro‒Wilk test. Differences among the three groups were calculated using one-way ANOVA followed by Tukey’s multiple comparisons for normally distributed samples and the Kruskal–Wallis test for data with non-Gaussian distribution. Unpaired *t*-test was used to calculate the significance between the two groups for normally distributed data and the Mann–Whitney *U* test for data with non-Gaussian distribution with the adjusted *p*-value < 0.05. The statistical analysis for RT-qPCR data was performed using GraphPad Prism 9.

### Supplementary Information

**Additional file 1: Figure S1.** vas::EGFP testicular cell transplantation into the sterile recipients. **Table S1. **Data from transplantation. **Figure S2. **Violin plots for methylation density in all three contexts. **Figure S3. **Correlation analysis for CG context. **Figure S4. **Venn plot for the number of DMRs in all the compared groups. **Figure S5. **Cluster heatmap for DMR methylation level. **Figure S6. **GO term enrichment between the compared groups. **Figure S7. **KEGG enrichment scatter plot for DMP-related pathway. **Table S2**. In vitro fertilization success of the germline chimeras and the donor. **Figure S8. **Images of the testis histological sections. **Figure S9. **EGFP expression in the sperm samples. **Figure S10. **Progenies derived from donor-derived sperm show no phenotypical alterations.

**Additional file 2. **The statistics of the genome coverage. File containing the genome coverage statistics for WGBS data.

**Additional file 3. **Summary of mapping results. File containing mapping results of the sequencing reads to the reference genome.

**Additional file 4. **The coverage statistics of cytosine in three different contexts. File containing the summary of the total percentage of cytosine bases covered in three different contexts.

**Additional file 5. **Summary of the DMR distribution in different genomic regions. File containing the distribution summary of the DMRs between the compared groups in different genomic regions.

**Additional file 6. **The number of significant DMPs in all the compared groups. File containing the number of significant DMPs in all the compared groups.

**Additional file 7. **Individual data values for qPCR. File containing relative gene expression data values for the individual samples.

**Additional file 8. **Gene ontology analysis of DMPs. File containing GO functional enrichment analysis of observed DMPs between all the compared groups.

**Additional file 9. **KEGG signaling pathway analysis of DMPs. File containing KEGG signaling pathway enrichment analysis of observed DMPs between all the compared groups.

**Additional file 10. **Primer sequences used in RT-qPCR. File containing the primer sequences used in RT-qPCR.

## Data Availability

The raw data files and the processed methylation data are publicly available at the Gene Expression Omnibus (GEO) under accession number GSE212876 [85]. Supplementary figures are provided in Additional file 1, and other data supporting the conclusions of this article are provided in Additional files 2, 3, 4, 5, 6, 7, 8, 9 and 10. All data generated or analysed during this study are included in this published article and its supplementary information files.

## Abbreviations

HybS: Sperm derived from the hybrid chimera
HybH: Progeny derived from the hybrid chimera
DoS: Sperm derived from the donor
DOH: Progeny derived from the donor
MOS: Sperm derived from the MO chimera
MOH: Progeny derived from the MO chimera
5-mC: 5- Methylcytosine
GSC: Germ stem cell
MO: Morpholino antisense nucleotide
PGC: Primordial gem cell
WGBS: Whole-genome bisulfite sequencing
GO: Gene Ontology
KEGG: Kyoto Encyclopedia of Genes and Genomes
TSS: Transcription start site
TES: Transcription end site
TE: Transposable element
DMP: Differentially methylated promoter
DMR: Differentially methylated region
GCT: Germ cell transplantation
IGV: Integrative Genomics Viewer
ML: Methylation level
mC: Methylated cytosine
MAPK: Mitogen-activated protein kinases
