# Supplementary material for: Genome-wide comparative methylation analysis reveals the fate of germ stem cells after surrogate production in teleost
Source: BMC Biol. 2024 Feb 16;22:39. doi: 10.1186/s12915-024-01842-z (PMC10870548; doi:10.1186/s12915-024-01842-z)
Supplement: Supplementary file 1 — Additional file 1: Figure S1. vas::EGFP testicular cell transplantation into the sterile recipients. Table S1. Data from transplantation. Figure S2. Violin plots for methylation density in all three contexts. Figure S3. Correlation analysis for CG context. Figure S4. Venn plot for the number of DMRs in all the compared groups. Figure S5. Cluster heatmap for DMR methylation level. Figure S6. GO term enrichment between the compared groups. Figure S7. KEGG enrichment scatter plot for DMP-related pathway. Table S2. In vitro fertilization success of the germline chimeras and the donor. Figure S8. Images of the testis histological sections. Figure S9. EGFP expression in the sperm samples. Figure S10. Progenies derived from donor-derived sperm show no phenotypical alterations. [file 12915_2024_1842_MOESM1_ESM.pdf]

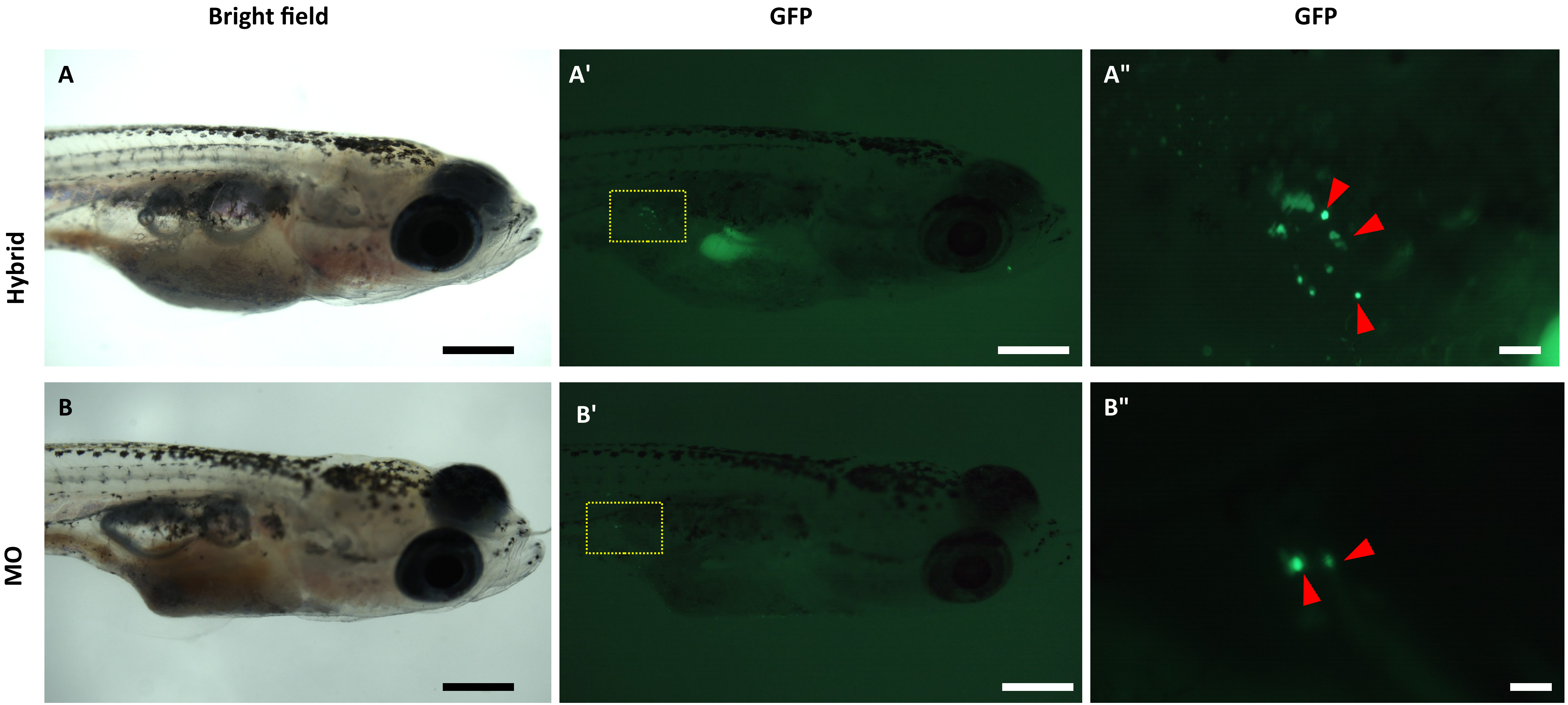

Figure S1. vas::EGFP testicular cell transplantation into the sterile recipients.
A) Two-weeks old transplanted hybrid recipient photographed under bright field seven-days post transplantation. **A'**) Same fish under fluorescence with the yellow color rectangle indicating few gfp-positive cells near the gas bladder (the region where gonad develops) from the donor origin, suggesting that cells are successfully incorporated into the recipient's gonad. **A**") Magnified area of the region depicted with yellow rectangle and cells are indicated by red arrowheads. **B**) The *dnd*-MO treated recipient of same age as hybrid under brightfield, **B**' and **B**") with two GFP-positive cells observed under fluorescence. scale bars – A, B, A ', and B ' = 1 mm, A" and B" = 100 µm.

Table S1. Data from transplantation

Replicate	Group	Total no. of transplanted recipient in each group	EGFP positive recipients (14 dpt)	Adult (in %)	Average adult (in %)
1	MO	58	23 (39.7 %)	36.0	40.4
2	MO	55	29 (52.7)	48.1	
3	MO	59	26 (44.1 %)	37.0	
1	Hybrid	47	18 (38.3%)	14.0	21.0
2	Hybrid	49	17 (34.7%)	28.6	
3	Hybrid	44	14 (31.8%)	20.5	

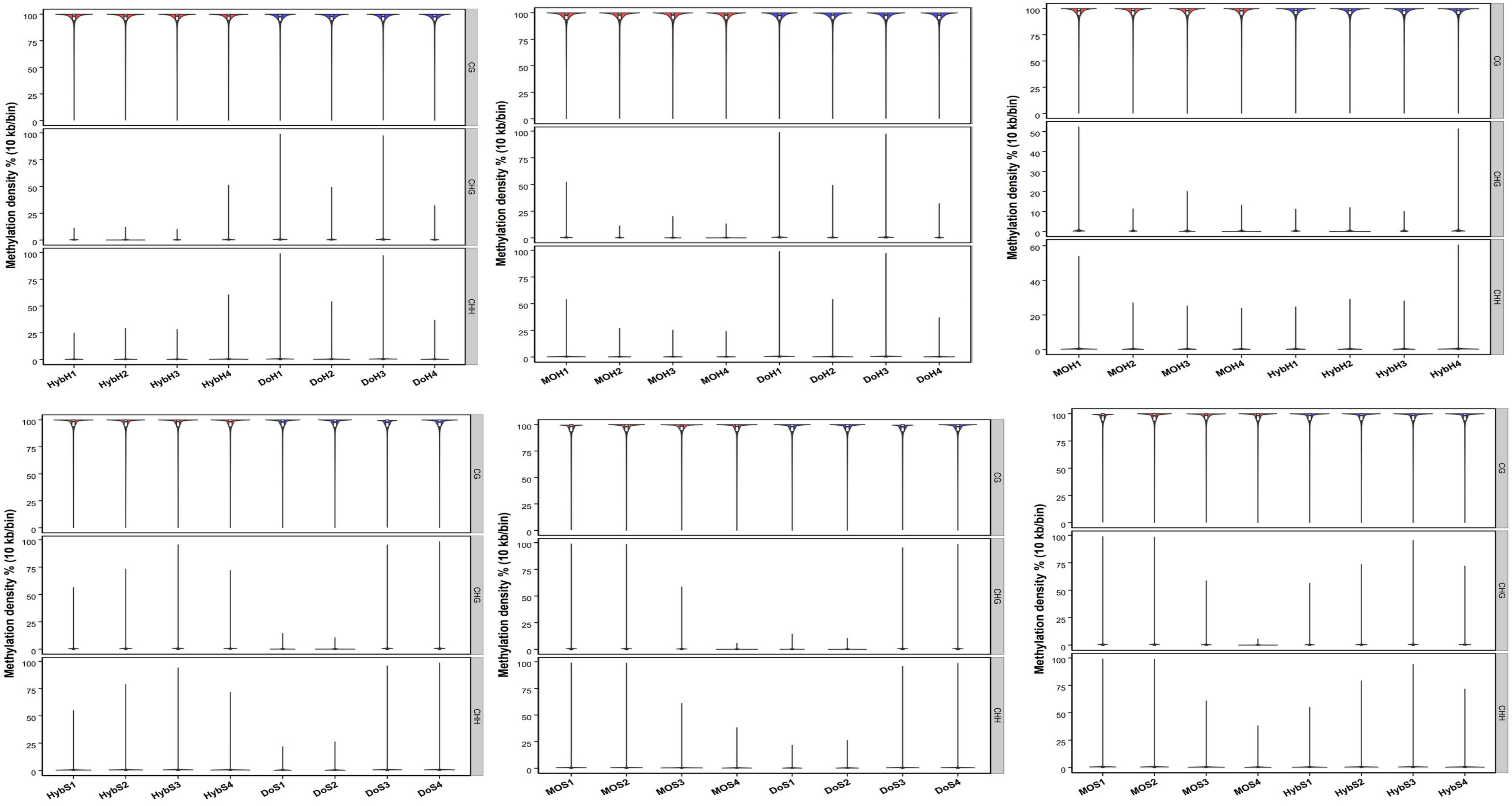

Figure S2. Violin plots for methylation density in all three context.
The whole genome was divided into the 10 kb sub-bin, and methylation density was calculated in each context. The x-axis indicates the name of each sample, the y-axis represent the methylation density.

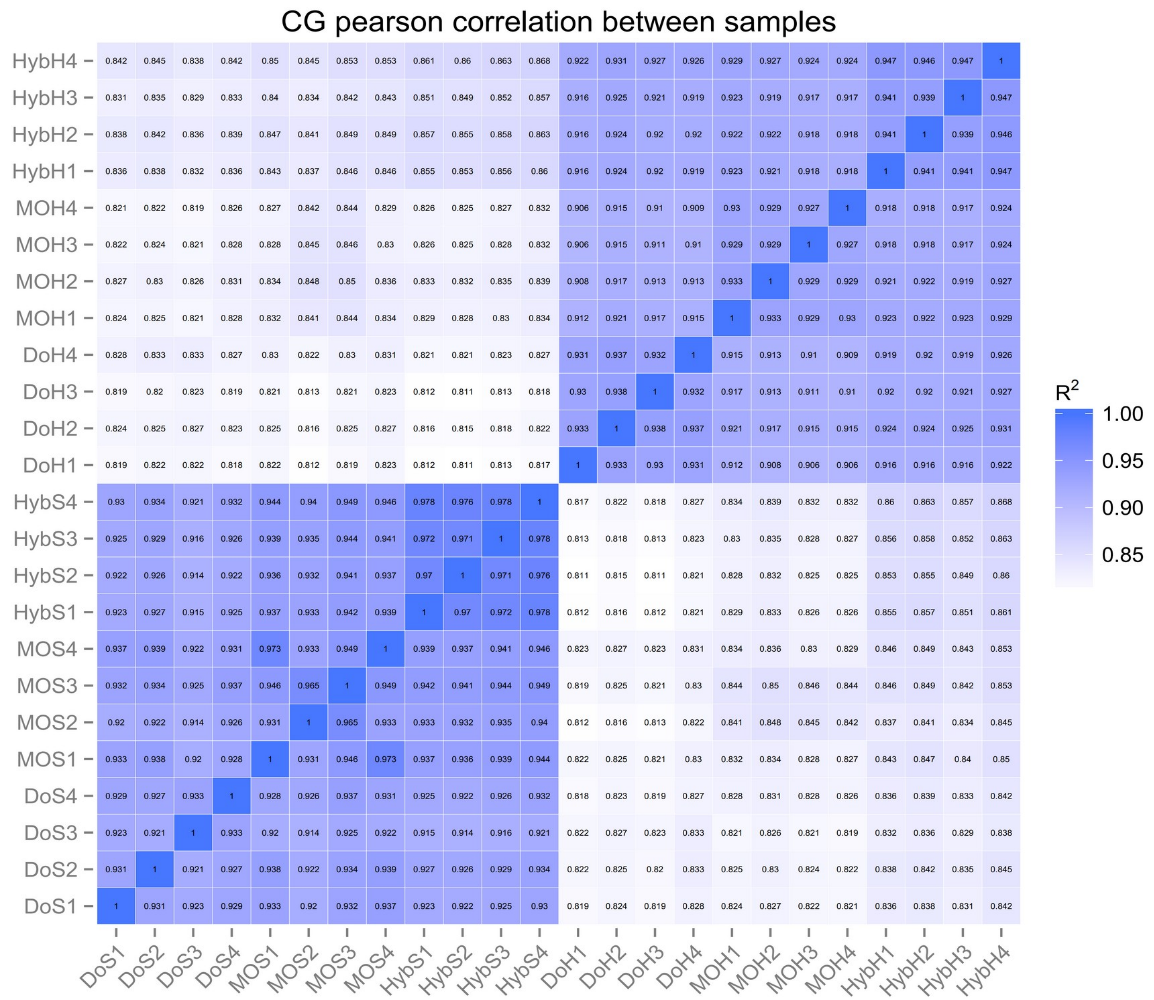

Figure S3. Correlation analysis for CG context.

The whole genome is splited into 2000 bp sub bin. The methylation level in each bin is calculated as follows: the number of mCs in the sub-bin/(the number of mCs in the sub-bin) + The number of non-mCs in the sub-bin. Heatmap represents the correlation between samples. R2- the square of Pearson correlation. HybS — sperm derived from the hybrid chimera; DoS — sperm derived from the donor; MOH — progeny derived from the MO-chimera; HybH — progeny derived from the hybrid chimera; and DoH — progeny derived from the donor, numbers 1, 2, 3 and 4 represents biological replicates in each group.

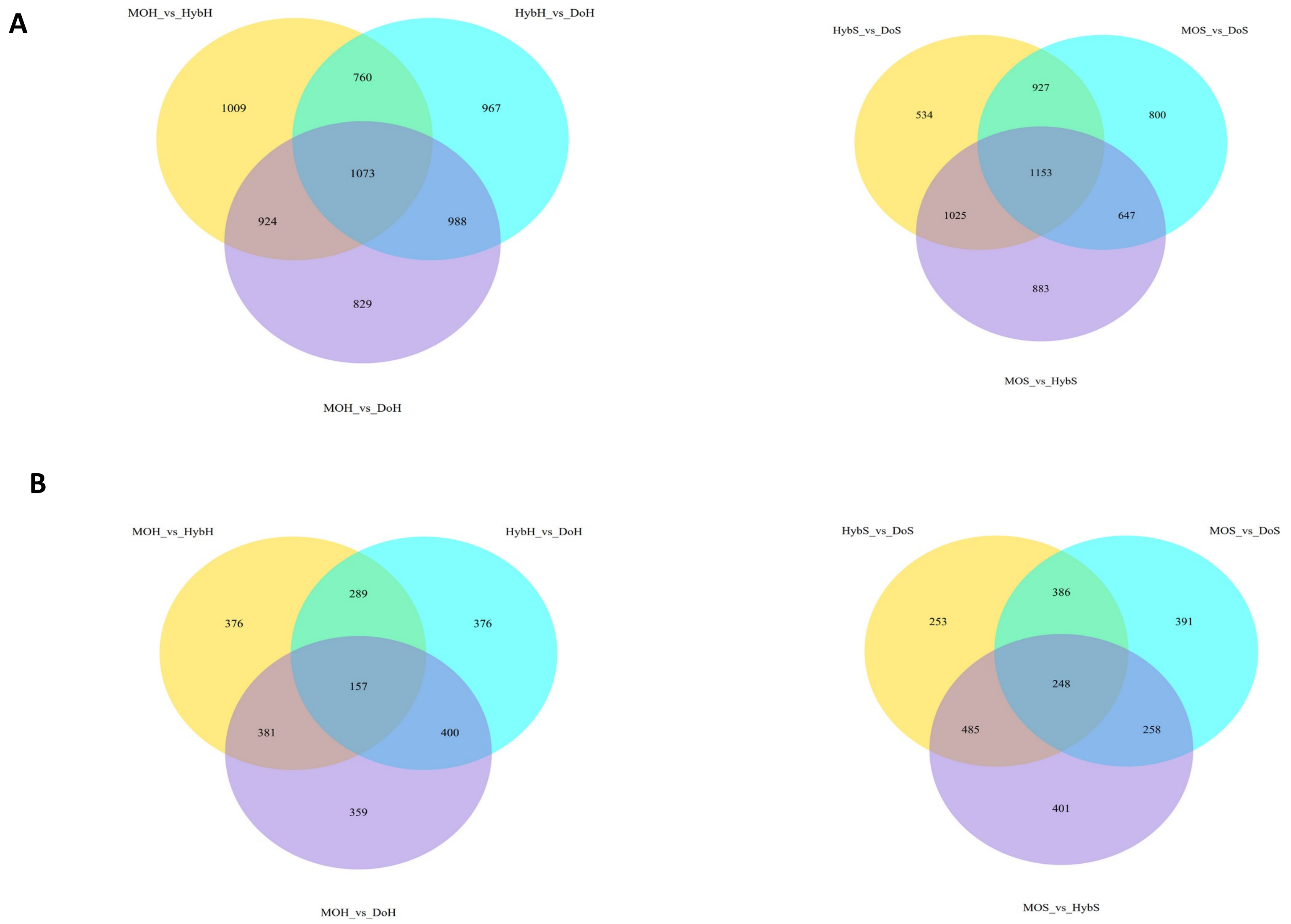

Figure S4. Venn plot for the number of DMRs in all the compared groups. **A**) Venn diagram for the progeny samples (left panel) and sperm samples (right panel). **B**) Number of DMPs in all the compared groups for the progeny samples (left panel) and sperm samples (right panel). The legend shows each comparison group, and the number indicates the number of genes in a group. The overlapping region is the number of genes shared among three comparison groups.

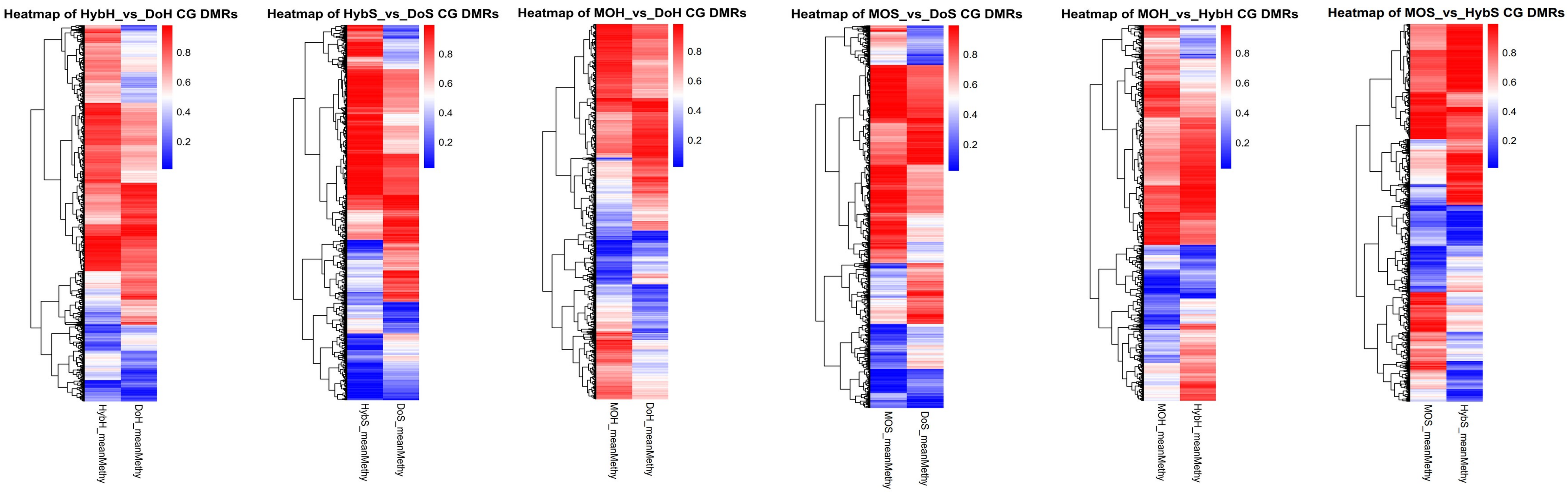

Figure S5. Cluster heatmap for DMR methylation level. The x-axis indicates the comparison group name, the y-axis represents the methylation level and cluster results.

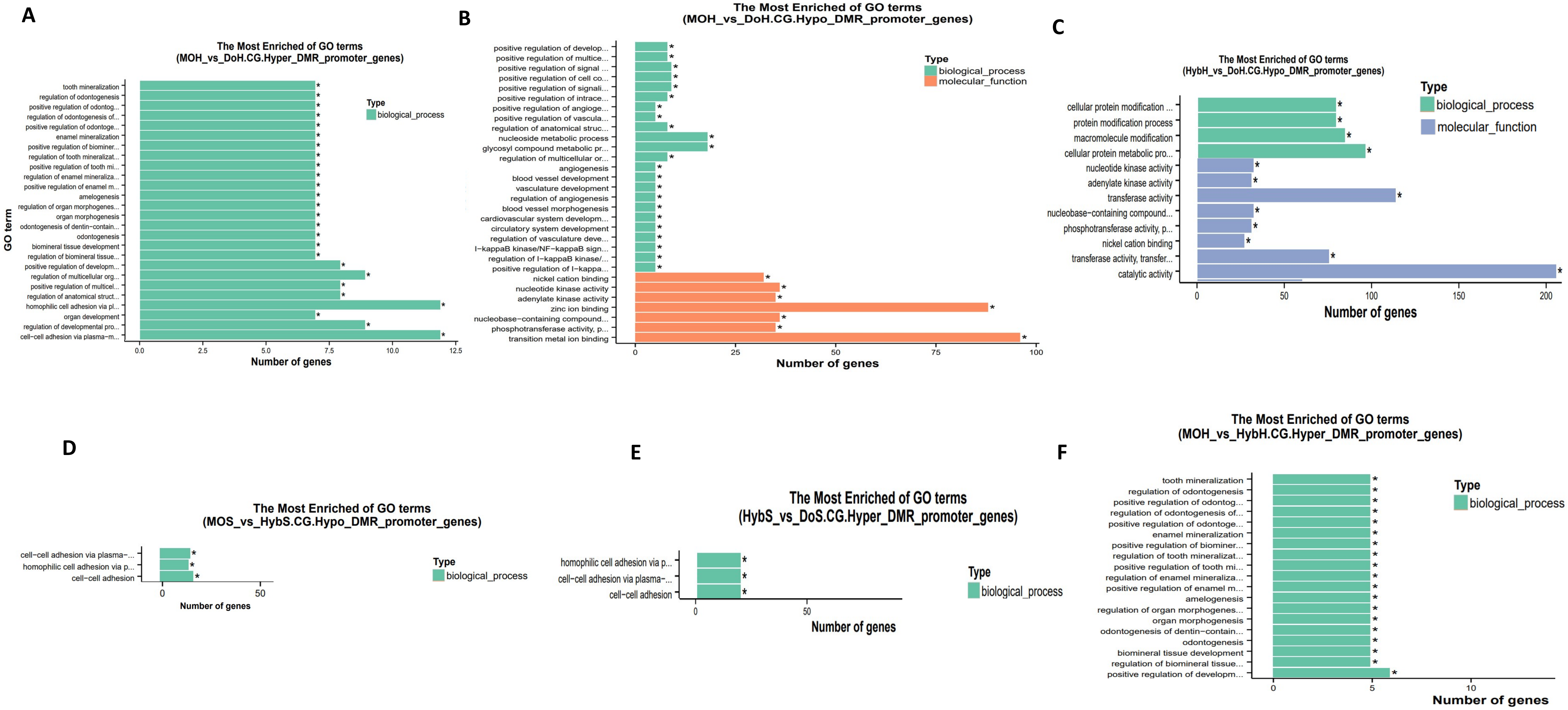

Figure S6. GO term enrichment between the compared groups. **A**) Enriched GO term between MOH and DOH for hyperDMPs, **B**) Enriched GO term between MOH and DOH for hypoDMPs. **C**) Enriched GO term between HybH and DOH for hypoDMPs, **D**) Enriched GO term between MOS and HybS for hypoDMPs, **E**) Enriched GO term between HybS and DOS for hyperDMPs, and **F**) Enriched GO term between MOH and HybH for hyperDMPs. The y-axis shows the enriched GO term, the x-axis shows the number of DMR-related genes, and colours denote three GO term categories: biological process, cellular component, and molecular function. GO terms with asterisks (*) are significantly enriched.

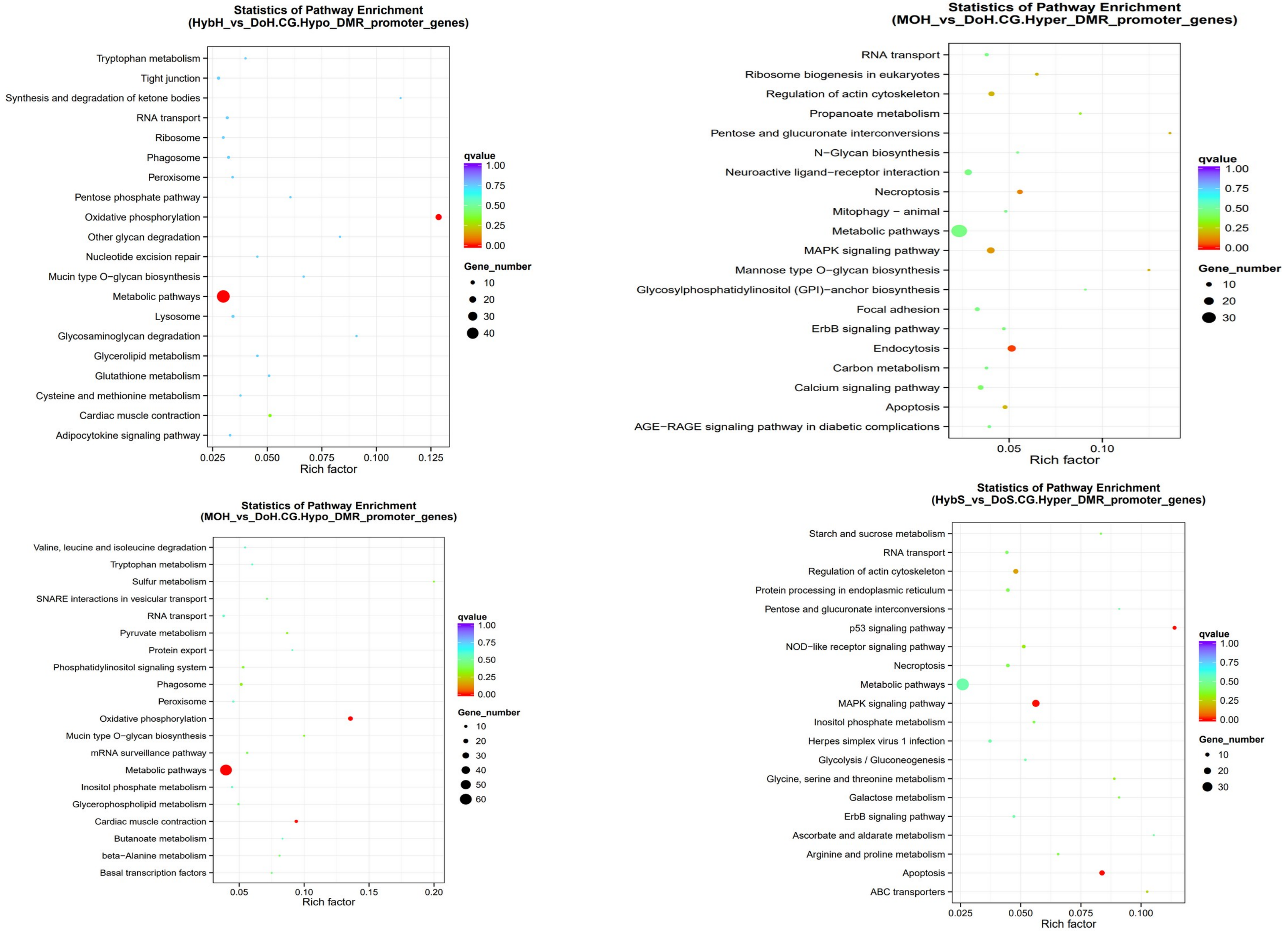

Figure S7. KEGG enrichment scatter plot for DMP-related pathway. The x-axis represents the rich factor, and the y-axis represents the pathway name. The size of points stands for DMP-related gene counts and the colours for different q-value ranges.

Table S2. In vitro fertilization success of the germline chimeras and the donor

Group	Total	segmentation	Hatched	Swim-up (5 dpf)
HybS1	100	45	42	42 (42%)
HybS2	100	32	32	32 (32%)
HybS3	100	39	39	39 (39%)
HybS4	100	41	40	42 (42%)
MOS1	100	89	89	89 (89%)
MOS2	100	86	86	86 (86%)
MOS3	100	92	92	92 (92%)
MOS4	100	87	87	87 (87%)
DOS1	100	95	95	95 (95%)
DOS2	100	92	92	92 (92%)
DOS3	100	97	97	96 (96%)
DOS4	100	97	97	93 (93%)

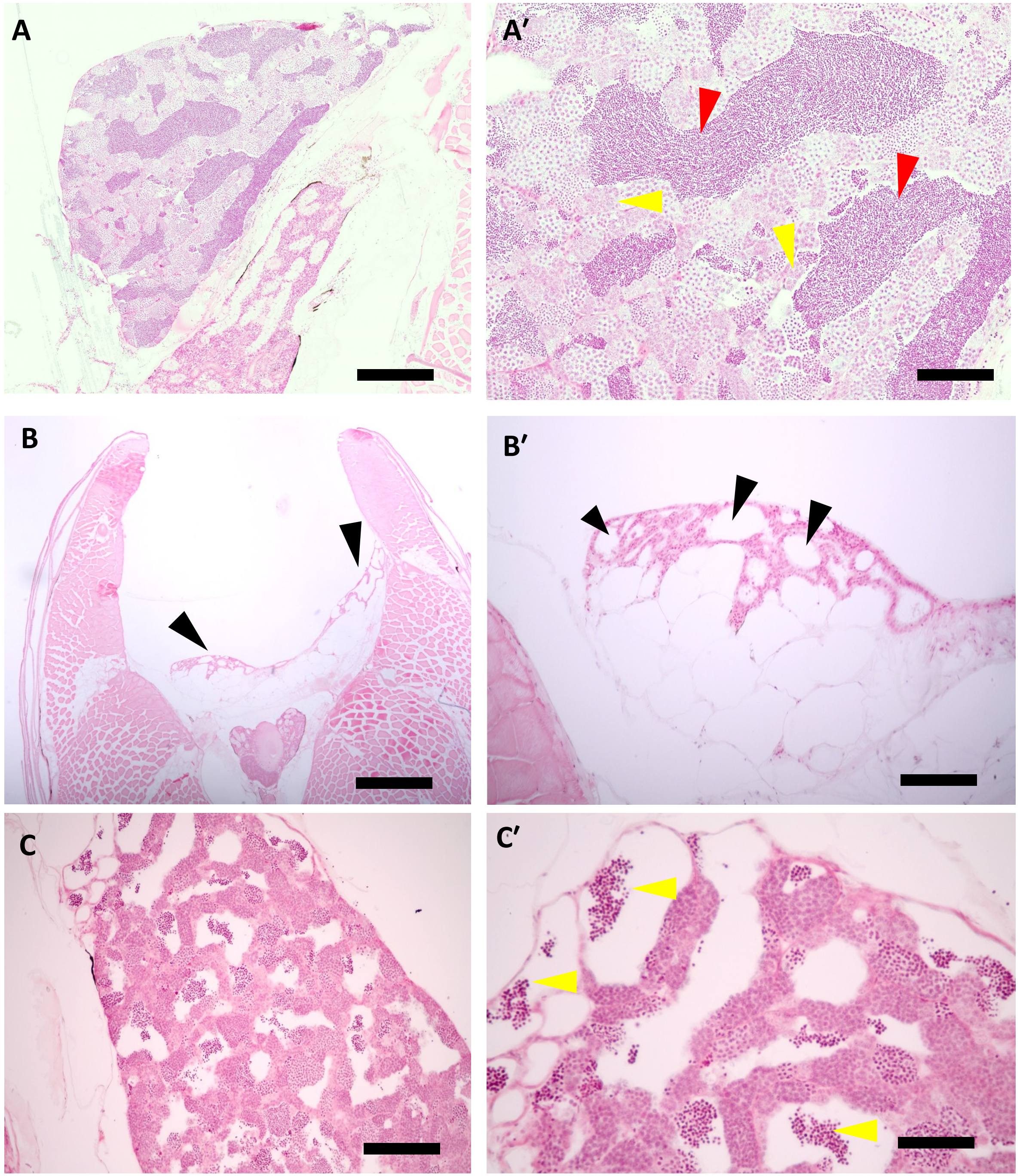

Figure S8. Images of the testis histological sections. A) Control male with well developed testis, A’) Higher magnification of the testis with red arrowheads indicates the spermatogonia and the yellow arrowheads indicates spermatozoa populations. B) Torso of the MO-treated recipient with black arrowhead indicating two underdeveloped gonad, thread testis-like structure, B’) The black arrowhead indicates empty lumen with lack of spermatozoa. C) Well developed testis of hybrid recipient, C’) The yellow arrowheads indicates very few spermatozoa in the lumen. scale bars, A, and C- 100 µm, A', B', C'= 50 µm, b= 200 µm.

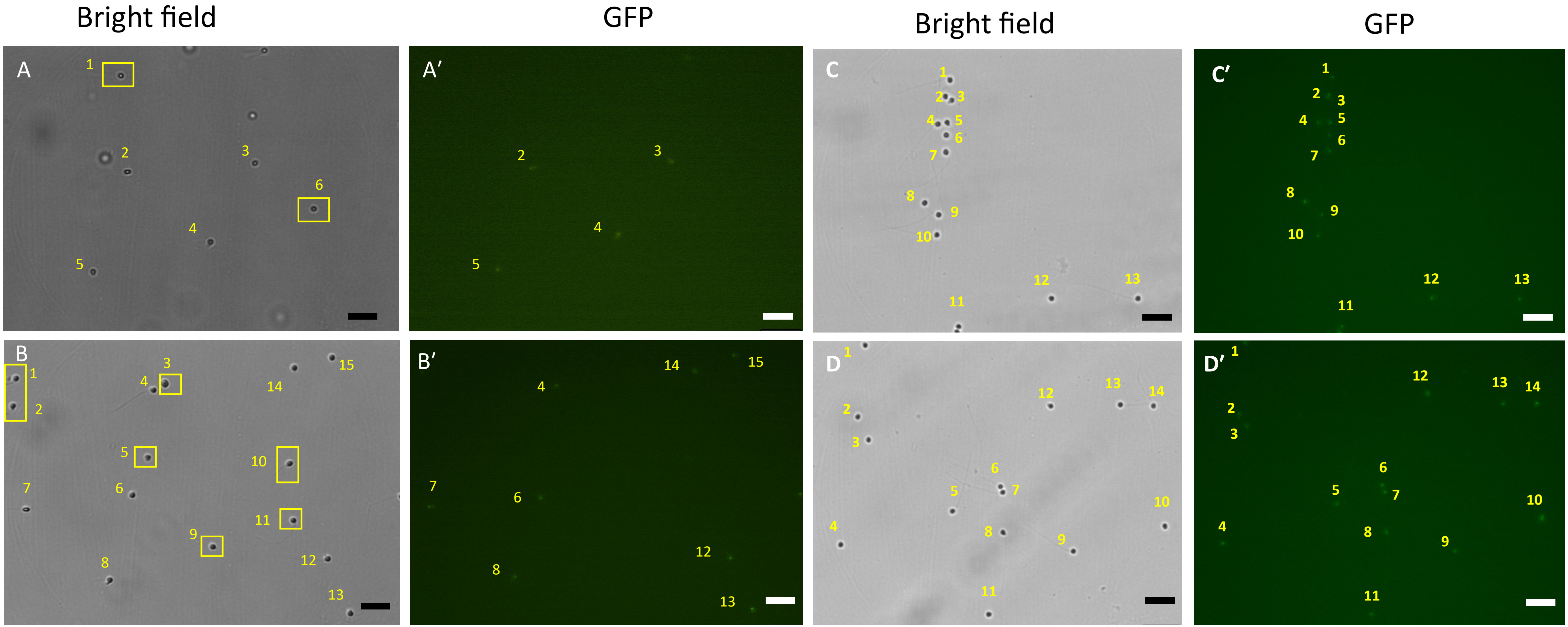

Figure S9. EGFP expression in the sperm samples. A)The donor-derived sperm samples from hybrid-chimera under bright field, the spermatozoa are numbered for better understanding, **A′) T**he same image was taken by GFP filter under fluorescence microscope (Olympus) and the number 1, and 6 spermatozoa (depicted by a yellow rectangle) do not have any signal under fluorescence for GFP. Similarly, **B**) The sperm from another hybrid-chimera under bright field, and **B**′) Fluorescence image of the same sample, here number 1, 2, 3, 5, 9, 10, and 11 have no signal for GFP. This results suggests that the semen sample from the hybris chimera is contaminated with recipient-derived spermatozoa. **C**) The donor-derived sperm sample from MO-chimera under bright field, and **C**′) The same samples under florescence with GFP expression in all spermatozoa. **D**) The sperm from another MO-chimera and **D**′) The signal for GFP is in all the spermatozoa, which confirms the presence of pure donor-derived spermatozoa. Scale bars = 20 µm.

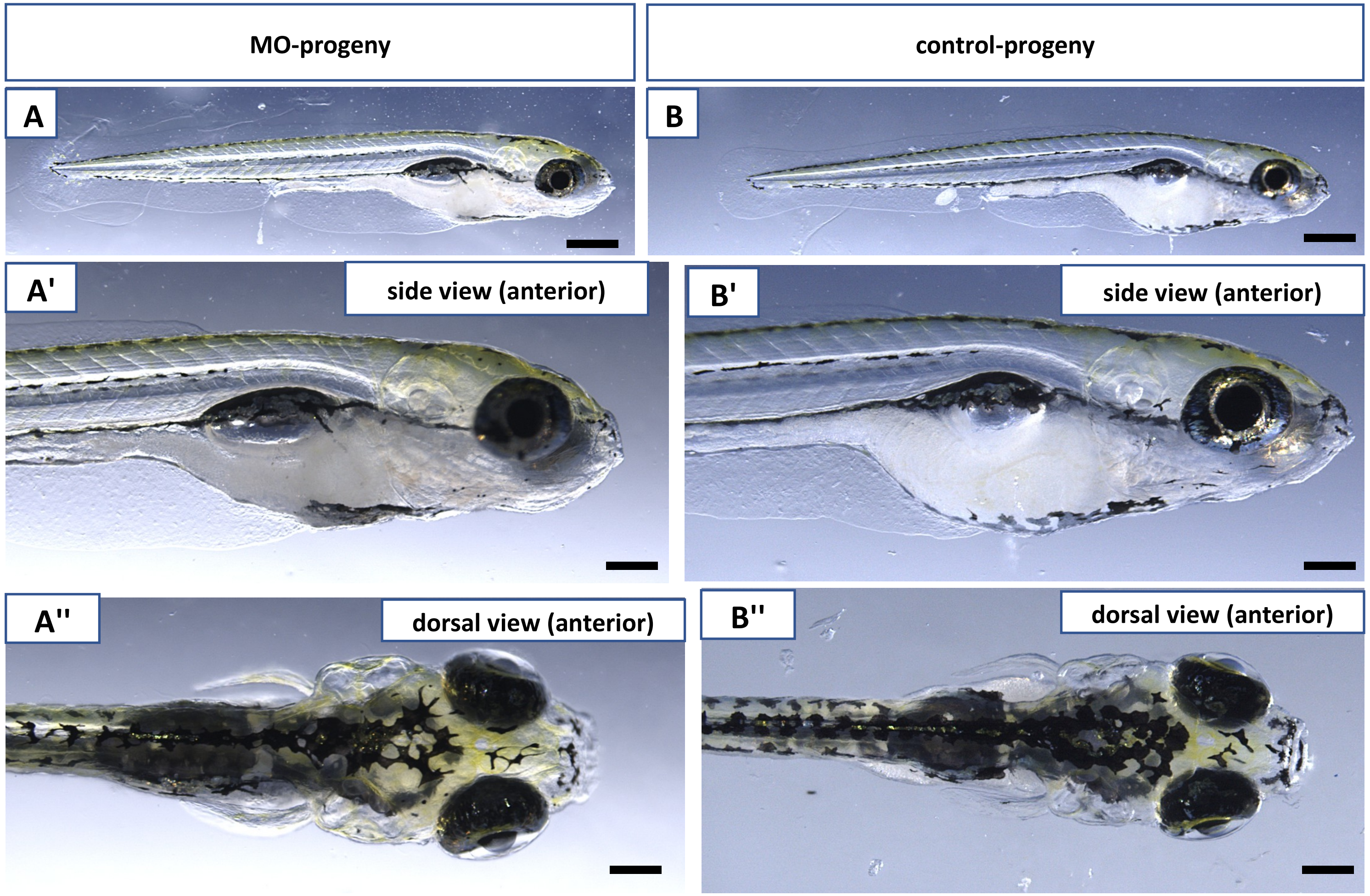

Figure S10. Progenies derived from the donor-derived sperm show no phenotypical alterations.
A-A“) Larvae (6 dpf) produced by in vitro fertilization of sperm from morpholino-treated chimera and the oocytes from the control female shows no morphological abnormalities. **B-B**“) Control group larvae produced by in vitro fertilization of donor sperm and oocyte from control female. scale bar, A and B = 1 mm, A’, A“, B’ and B“ = 100 µm.
